# Efficacy and Safety of Gemcitabine With Trastuzumab and Pertuzumab After Prior Pertuzumab-Based Therapy Among Patients With Human Epidermal Growth Factor Receptor 2–Positive Metastatic Breast Cancer: A Phase 2 Clinical Trial

**DOI:** 10.1001/jamanetworkopen.2019.16211

**Published:** 2019-11-27

**Authors:** Neil M. Iyengar, Lillian M. Smyth, Diana Lake, Ayca Gucalp, Jasmeet C. Singh, Tiffany A. Traina, Patricia DeFusco, Monica N. Fornier, Shari Goldfarb, Komal Jhaveri, Shanu Modi, Tiffany Troso-Sandoval, Sujata Patil, Gary A. Ulaner, Maxine Jochelson, Larry Norton, Clifford A. Hudis, Chau T. Dang

**Affiliations:** 1 Memorial Sloan Kettering Cancer Center, New York, New York; 2 Weill Cornell Medicine, New York, New York

## Abstract

**Importance:**

Taxanes with trastuzumab and pertuzumab for initial treatment of human epidermal growth factor receptor 2 (*ERBB2*, formerly *HER2*)–positive metastatic breast cancer is associated with improved progression-free survival (PFS) and overall survival. While continued use of trastuzumab in therapeutic combinations after disease progression is standard, the efficacy of continuing pertuzumab is unknown.

**Objective:**

To evaluate the efficacy and safety of pertuzumab in combination with gemcitabine and trastuzumab after prior treatment with pertuzumab for *ERBB2*-positive metastatic breast cancer.

**Design, Setting, and Participants:**

This is a phase 2 single-arm clinical trial of dual anti-*ERBB2* therapy after prior treatment with pertuzumab. The study took place at a single academic center from March 2015 to April 2017 among women with *ERBB2*-positive metastatic breast cancer, prior pertuzumab-based treatment, and 3 or fewer prior chemotherapy regimens. Data were analyzed between January 2019 and March 2019.

**Intervention:**

Treatment consisted of gemcitabine, 1200 mg/m^2^ (later amended to 1000 mg/m^2^) on days 1 and 8 every 3 weeks, plus trastuzumab (8-mg/kg loading dose, then 6 mg/kg) and pertuzumab (840-mg loading dose, then 420 mg) once every 3 weeks.

**Main Outcomes and Measures:**

The primary end point was 3-month PFS. Based on prior trials, a target rate of 70% or higher was selected as the promising progression-free rate at 3 months. Secondary outcomes included safety, tolerability, and overall survival.

**Results:**

A total of 45 patients (median [range] age, 57.1 [31.7-77.2] years) were enrolled; 22 (49%) were treated in the second-line setting, and 23 (51%) were treated in the third-line setting or beyond. Of these, 22 (49%) received prior trastuzumab emtansine (T-DM1). At a median (range) follow-up of 27.6 (8.3-36.0) months, 3-month PFS was 73.3% (95% CI, 61.5%-87.5%). Overall, median PFS was 5.5 months (95% CI, 5.4-8.2 months). Treatment was well tolerated, with no occurrences of febrile neutropenia or symptomatic left ventricular systolic dysfunction.

**Conclusions and Relevance:**

In this phase 2 trial, treatment with gemcitabine, trastuzumab, and pertuzumab after prior pertuzumab-based therapy for *ERBB2*-positive metastatic breast cancer was associated with a 3-month PFS rate of 73.3% and was well tolerated. Continuation of pertuzumab beyond progression was associated with apparent clinical benefit.

**Trial Registration:**

ClinicalTrials.gov identifier: NCT02252887

## Key Points

**Question:**

Is dual anti–human epidermal growth factor receptor 2 (*ERBB2, *formerly *HER2*) therapy with trastuzumab and pertuzumab effective after prior pertuzumab-based therapy for *ERBB2*-positive metastatic breast cancer?

**Findings:**

In this phase 2 clinical trial, treatment with gemcitabine, trastuzumab, and pertuzumab after prior pertuzumab-based therapy for *ERBB2*-positive metastatic breast cancer was associated with a 3-month progression free survival rate of 73.3%. Treatment was well tolerated with no occurrences of symptomatic left ventricular systolic dysfunction.

**Meaning:**

In this study, dual anti-*ERBB2* therapy with trastuzumab and pertuzumab after prior pertuzumab exposure was active and well tolerated.

## Teaser

This phase 2 clinical trial evaluates the efficacy and safety of pertuzumab in combination with gemcitabine and trastuzumab after prior treatment with pertuzumab for human epidermal growth factor receptor 2 (*ERBB2*, formerly *HER2*)–positive metastatic breast cancer.

## Introduction

Human epidermal growth factor receptor 2 (*ERBB2*, formerly *HER2*) is a proto-oncogene that encodes the 185-kDa ERBB2 protein, a transmembrane tyrosine kinase receptor that is a member of the human epidermal growth factor receptor family. Human epidermal growth factor receptor 2, which is involved in the regulation of cell growth and survival, is amplified in 15% to 20% of invasive breast carcinomas and confers shortened progression-free survival (PFS) and overall survival (OS) compared with other breast tumors.^1^ Activation of ERBB signaling is dependent on homodimerization or heterodimerization, with the ERBB2-ERBB3 dimer as the most potent inducer of cell proliferation.^2^ Clinical outcomes for patients with *ERBB2*-positive breast cancer have significantly improved with the development of ERBB2-targeted therapies. Trastuzumab is a humanized monoclonal antibody that binds to the extracellular domain IV of ERBB2 and thereby limits its activation.^3^ Pertuzumab, also a monoclonal antibody, disrupts ERBB2 dimerization via binding of the extracellular domain II of ERBB2, thereby inhibiting ligand-activated signaling with other growth factor receptors.^4^ Together, trastuzumab and pertuzumab synergistically inhibit tumor growth through their complementary mechanisms of action.^4^

In the frontline setting, standard treatment of patients with *ERBB2*-positive metastatic breast cancer includes the combination of taxane chemotherapy with trastuzumab and pertuzumab.^5,6^ This approach was established by pivotal data from the randomized phase 3 Clinical Evaluation of Trastuzumab and Pertuzumab (CLEOPATRA) trial,^5^ which demonstrated improvements in both PFS and OS in patients treated with docetaxel, trastuzumab, and pertuzumab on a 3-week schedule. Our group reported similar findings in a phase 2 study^6^ evaluating the combination of weekly paclitaxel with trastuzumab and pertuzumab. The use of either docetaxel or weekly paclitaxel with trastuzumab and pertuzumab for first-line treatment of patients with *ERBB2*-positive metastatic breast cancer is endorsed by the National Comprehensive Cancer Network guidelines.^7^

In the second-line setting, the randomized phase 3 PHEREXA trial^8^ evaluated the combination of capecitabine with trastuzumab and pertuzumab vs capecitabine with trastuzumab alone. While the addition of pertuzumab in the second-line setting was not associated with a significant gain in PFS, an 8-month increase in OS was reported, although the trial was underpowered to evaluate this end point. Other trials have demonstrated median PFS of approximately 3 to 4 months for patients treated with trastuzumab and chemotherapy-based regimens after 2 or more prior lines of therapy.^9,10,11^ Based on these historical data, we aimed to determine the 3-month and median PFS rates of dual anti-*ERBB2* therapy, including pertuzumab and trastuzumab, given with chemotherapy among patients previously exposed to pertuzumab. Accordingly, we conducted a phase 2 trial of gemcitabine with pertuzumab and trastuzumab in patients with *ERBB2*-positive metastatic breast cancer in the second-line setting and beyond.

## Methods

### Study Population and Design

This was a single-center, phase 2 trial in which all patients were enrolled from the Memorial Sloan Kettering Cancer Center. Eligible patients had *ERBB2*-positive metastatic breast cancer and prior treatment with pertuzumab-based therapy. We defined *ERBB2* positivity as 3+ by immunohistochemistry stain or amplified by fluorescent in situ hybridization with an *ERBB2* to *CEP17* ratio of 2.0 or greater. Additional eligibility criteria included being 18 years or older, having an Eastern Cooperative Oncology Group performance status of 0 to 1, having measurable or nonmeasurable disease, having had up to 3 prior chemotherapy regimens in the metastatic setting, having adequate organ function, and having baseline left ventricular ejection fraction (LVEF) of 50% or greater, measured by echocardiogram, within 4 weeks before enrollment. Patients may have had pertuzumab-based therapy in the neoadjuvant or adjuvant setting. Patients with treated brain metastasis who were stable for 2 or more months before enrollment were included. Patients were excluded if they had a history of cardiac morbidities (eg, unstable angina, myocardial infarction, congestive heart failure, or uncontrolled ventricular arrhythmias) within 12 months of enrollment or any grade 3 or higher toxic response to prior trastuzumab or pertuzumab. This trial was approved by the institutional review board of the Memorial Sloan Kettering Cancer Center, and all participants provided written informed consent prior to enrollment. Reporting of this trial is in accordance with Consolidated Standards of Reporting Trials (CONSORT) reporting guideline. The clinical trial protocol is available as Supplement 1.

### Treatment

Patients received gemcitabine at 1200 mg/m^2^ on days 1 and 8 of a 21-day cycle and trastuzumab (8-mg/kg loading dose, then 6 mg/kg) plus pertuzumab (840-mg loading dose, then 420 mg) once every 3 weeks, all given intravenously (Table 1). Each cycle spanned 3 weeks and included gemcitabine with trastuzumab and pertuzumab given on day 1 and gemcitabine alone on day 8. After 3 months of therapy, if patients were deemed to be progression free, gemcitabine could be held at the discretion of the treating physician, and patients were maintained with antibodies alone. Treatment was continued until progression of disease or unacceptable toxic effects.

**Table 1.** Treatment Schema

Medication	Dosage	Frequency^a^	
		Day 1	Day 8
Gemcitabine	1000 mg/m^2^	X	X
Pertuzumab	840-mg loading dose, then 420 mg	X	
Trastuzumab	8-mg/kg loading dose, then 6 mg/kg	X	

^a^ Medication was given on a 21-day cycle.

### Treatment Modifications

For grade 3 or greater toxic effects, gemcitabine was held until the adverse event (AE) improved to grade 2 or less. Up to 3 consecutive weeks of treatment delay was allowed for recovery. On improvement and reinitiation of treatment, the gemcitabine dose was reduced. A maximum of 2 dose reductions were permitted (ie, from 1200 mg/m^2^ to 1000 mg/m^2^ and from 1000 mg/m^2^ to 800 mg/m^2^; later amended to 1000 mg/m^2^ to 800 mg/m^2^ and from 800 mg/m^2^ to 600 mg/m^2^). For trastuzumab and pertuzumab, treatment was held for significant asymptomatic LVEF decline (ie, 10%-15% decline to <50%, or ≥10% decline from baseline) or New York Heart Association class III to class IV heart failure. Treatment with the anti-*ERBB2* antibodies could be restarted if repeated echocardiogram showed LVEF recovery within 3 weeks. Trastuzumab or pertuzumab dose reductions were not permitted.

### Clinical Assessments

Response to therapy was evaluated with serial computed tomography scan of the chest, abdomen, and pelvis every 3 months during study treatment using the Response Evaluation Criteria in Solid Tumors (RECIST) version 1.1.^12^ Patients also received optional fludeoxyglucose F 18 positron emission tomography–computed tomography scans every 3 months, and an exploratory end point was to evaluate response by Positron Emission Tomography Response Criteria in Solid Tumors (PERCIST) version 1.0.^13^ A complete blood cell count was obtained before each chemotherapy dose. Patients were seen once per cycle, with comprehensive chemistry laboratory assessments performed every cycle. Monitoring for LVEF occurred at baseline and every 3 months within 3 months after treatment completion by echocardiogram or multigated acquisition study. Monitoring for AEs occurred continuously, and toxic effects were graded according to the National Cancer Institute’s Common Terminology Criteria for Adverse Events, version 4.0.

### Statistical Analysis

The primary end point of this study was the proportion of patients who were progression free at 3 months or later. Progression-free survival events included disease progression or death, whichever occurred first. The study was powered on the basis of a binary end point of the proportion of patients who were progression free at 3 months. For the primary intention-to-treat analysis, evaluable patients included all study participants who received at least 1 full dose of therapy. A target rate of 70% or higher was selected as the promising progression-free rate at 3 months, while the null rate of 50% was based on evidence from 3 prior studies involving more than 900 patients with *ERBB2*-positive metastatic breast cancer treated in the second-line setting or beyond, in which median PFS was 3 to 4 months.^9,10,11^ A Simon optimal 2-stage design was used to evaluate the efficacy of this regimen.^14^ This design required a total of 45 patients and assumed a 10% type I and type II error rate. The type I error was 1-sided, and secondary analyses were conducted at a 2-sided type I error of 5%. In the first stage, 21 patients were enrolled. Accrual to the second stage was contingent on 12 or more patients in the first stage being alive and progression free at 3 months. At the end of the study, if 27 or more patients were alive and progression free 3 months after the initiation of therapy, the regimen would be considered a success and deemed worthy of further study. The Kaplan-Meier method was used to calculate PFS estimates. A secondary end point was OS, which was estimated using the Kaplan-Meier method. Additional secondary end points included safety, tolerability, and assessment of biomarkers (not presented in this article). An exploratory end point was to evaluate response by PERCIST (not presented in this article). Adverse events with frequencies of 25% or greater were summarized by percentage. We incorporated a stopping rule to ensure cardiac safety. Cardiac events were defined as symptomatic left ventricular systolic dysfunction (LVSD; deaths and nondeaths), non-LVSD cardiac death, or probable cardiac death.^15^ A cardiac event rate of 4% or less and an asymptomatic LVEF decline (of ≥10% from baseline to <50%) rate of 20% or less were considered acceptable. Data were analyzed between January 2019 and March 2019. All analyses were conducted using SAS statistical software version 9.2 (SAS Institute) and R version 3.5.0 (R Project for Statistical Computing).

## Results

### Study Population

A total of 45 patients were enrolled from March 2015 to April 2017. Baseline characteristics are presented in Table 2. The median (range) age was 57.1 (31.7-77.2) years. At initiation of study treatment, 38 patients (84%) had visceral disease, and 7 (16%) had nonvisceral disease. Measurable disease was present in 30 patients (67%), and 15 (33%) had nonmeasurable disease. A total of 33 patients (73%) had estrogen receptor–positive and/or progesterone receptor–positive disease. Metastatic disease was present at the time of breast cancer diagnosis in 20 patients (44%). Overall, 22 patients (49%) had received 1 prior line of chemotherapy in the metastatic setting, 17 (38%) received 2 prior lines, and 6 (13%) received 3 prior lines. Notably, 22 patients (49%) received prior trastuzumab emtansine (T-DM1).

**Table 2.** Baseline Characteristics for 45 Patients

Characteristic	No. (%)
Age, median (range), y	57.1 (31.7-77.2)
Race	
Asian	4 (9)
Black	6 (13)
White	32 (71)
Unknown	3 (7)
ECOG PS	
0	30 (67)
1	15 (33)
Disease type at screening	
Nonvisceral	7 (16)
Visceral	38 (84)
Measurable disease	30 (67)
Nonmeasurable disease	15 (33)
Hormone receptor status	
ER and/or PgR positive	33 (73)
ER and PgR negative	12 (27)
*ERBB2* status assessed by IHC	
0 or 1+	5 (11)
2+	15 (33)
3+	24 (53)
Unknown	1 (2)
*ERBB2* status assessed by FISH	
Amplified	25 (56)
Not amplified	1 (2)
Not assessed	19 (42)
Metastatic disease at diagnosis	20 (44)
Prior lines of chemotherapy-based treatment for metastatic disease	
1	22 (49)
2	17 (38)
3	6 (13)
Prior treatment with T-DM1	22 (49)
Prior adjuvant or neoadjuvant therapy	
No	22 (49)
Yes	23 (51)
Anthracycline	14 (31)
Taxane	18 (40)
Trastuzumab	18 (40)
Pertuzumab	2 (4)
Other	1 (2)
Unknown	1 (2)

Abbreviations: ECOG PS, Eastern Cooperative Oncology Group performance status; ER, estrogen receptor; FISH, fluorescent in situ hybridization; IHC, immunohistochemistry; PgR, progesterone receptor; T-DM1, trastuzumab emtansine.

### Efficacy

Of the 45 enrolled patients, 1 was not evaluable because she received concurrent endocrine therapy with study treatment. Accordingly, 44 patients were included in efficacy analyses (eFigure in Supplement 2). At a median (range) follow-up period of 27.6 (8.3-36.0) months, the 3-month PFS was 73.3% (95% CI, 61.5%-87.5%). The 3-month PFS was 73.9% (95% CI, 58.0%-94.2%) in patients who had not received prior T-DM1 and 72.7% (95% CI, 56.3%-93.9%) in those who received prior T-DM1. Overall, median PFS was 5.5 months (95% CI, 5.4-8.2 months) (Figure, A); it was 5.6 months (95% CI, 5.1-11.0 months) for those without prior T-DM1 treatment and 5.5 months (95% CI, 5.4-8.2 months) for those who received prior T-DM1 (Figure, B). The 3-month OS rate was 100%, and median OS was not reached (Figure, C). In terms of clinical benefit, 1 patient (2%) had a complete response, 9 (20%) had a partial response, and 23 (52%) had stable disease (Table 3).

**Figure.**
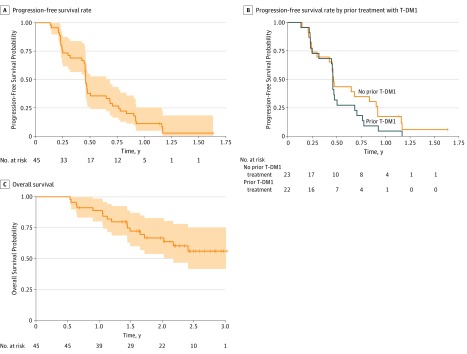
Kaplan-Meier Estimates of Progression-Free and Overall Survival T-DM1 indicates trastuzumab emtansine. Shaded areas (A and C) indicate 95% CIs.

**Table 3.** Efficacy at 3 Months for 44 Patients^a^

Status	No. (%)
Progression free	33 (75)
CR	1 (2)
PR	9 (20)
Stable disease	13 (30)
Non-CR or non-PR	10 (23)
Progression of disease	11 (25)

Abbreviations: CR, complete response; PR, partial response.

^a^ In all, 44 patients were eligible for 3-month response assessment.

### Treatment Exposure

The median (range) duration of study treatment was 20.8 (1.6-35.6) months for all enrolled patients. The median (range) number of study treatment cycles per patient was 8 (3-37). The median (range) number of cycles of gemcitabine exposure was 8 (3-31). Initial gemcitabine recommended dose was 1200 mg/m^2^, which was delivered to 5 patients. Owing to significant toxic effects, described in the next section, the study was amended to deliver gemcitabine at 1000 mg/m^2^ on days 1 and 8 every 3 weeks; this was also the median dose intensity. The median (range) number of cycles of dual-antibody exposure was 8 (3-37).

### Toxic Effects Profile and Cardiac Safety

Within the first 3 months of the study, 5 patients required gemcitabine dose reduction (4 patients because of grade 3 neutropenia and 1 patient because of grade 3 vomiting). Accordingly, the study was amended to lower the initial dose of gemcitabine from 1200 mg/m^2^ to 1000 mg/m^2^. At this dose, gemcitabine with trastuzumab and pertuzumab was well tolerated with no unexpected AEs (Table 4). Grade 3 or higher AEs included neutropenia (23 patients [51%]), anemia (6 patients [13%]), alanine aminotransferase level elevation (2 patients [4%]), aspartate transaminase level elevation (1 patient [2%]), fatigue (1 patient [2%]), thrombocytopenia (1 patient [2%]), diarrhea (1 patient [2%]), and nausea (1 patient [2%]). Notably, the incidence of febrile neutropenia was 0%.

**Table 4.** Adverse Events^a^

Adverse Event	Grade, No. (%)^b^			
	1	2	3	4
Fatigue	35 (78)	6 (13)	1 (2)	0
Anemia	16 (36)	17 (38)	6 (13)	0
Neutropenia	1 (2)	8 (18)	21 (47)	2 (4)
Thrombocytopenia	25 (56)	3 (7)	1 (2)	0
Peripheral neuropathy	24 (53)	5 (11)	0	0
ALT level elevation	23 (51)	2 (4)	2 (4)	0
AST level elevation	19 (42)	3 (7)	1 (2)	0
Diarrhea	17 (38)	3 (7)	1 (2)	0
Nausea	16 (36)	4 (9)	1 (2)	0
Alkaline phosphatase level elevation	16 (36)	2 (4)	0	0
Cough	15 (33)	1 (2)	0	0
Myalgia	12 (27)	1 (2)	0	0
Arthralgia	12 (27)	0	0	0
Dyspnea	9 (20)	2 (4)	0	0

Abbreviations: ALT, alanine aminotransferase; AST, aspartate transaminase.

^a^ Includes all patients who received at least 1 dose of study treatment.

^b^ Adverse events of all grades shown have a frequency of 25% or higher.

Regarding cardiac safety, median LVEF was preserved throughout. No patients experienced protocol-defined cardiac events (symptomatic LVSD). The median (range) LVEF was 60% (52%-80%) at baseline and 61% (45%-70%) at 3 months. One patient (2%) with no known cardiac history had an asymptomatic LVEF decline. She met the eligibility criteria for study enrollment with an LVEF of 57% at baseline. At 3 months, her LVEF decreased to 45%, at which point dual-antibody therapy was held. A repeated echocardiogram was performed 2 weeks later, which showed that her LVEF had recovered to 58% and dual-antibody therapy was resumed. A follow-up echocardiogram 4 weeks later showed preserved LVEF at 55%.

## Discussion

In this study of gemcitabine given with dual anti-*ERBB2* antibody therapy for the treatment of *ERBB2*-positive metastatic breast cancer among patients previously treated with pertuzumab-based therapy, the Kaplan-Meier 3-month PFS was 73.3% (95% CI, 61.5%-87.5%). This study met its primary end point and prespecified definition of success: a promising 3-month PFS rate of at least 70%. Thus, this trial demonstrated encouraging activity of dual anti-*ERBB2* therapy with gemcitabine in the second-line setting and beyond.

Nearly half of patients in this study received T-DM1 after disease progression on first-line treatment with taxane plus dual anti-*ERBB2* therapy. Currently, T-DM1 is a standard second-line treatment based on the EMILIA study^16^ and endorsed by American Society of Clinical Oncology guidelines^17^ for systemic therapy in patients with *ERBB2*-positive metastatic breast cancer. The EMILIA trial, a randomized phase 3 study,^16^ demonstrated improved PFS (9.6 vs 6.4 months) and OS (29.9 vs 25.9 months) with T-DM1 compared with the combination of lapatinib plus capecitabine.^18^ With the negative results of the first-line MARIANNE study,^19^ T-DM1 remains a standard option in the second-line setting. However, our data encourage further study of reexposure to trastuzumab plus pertuzumab beyond progression from prior treatment with this dual-antibody therapy. Retrospective data from our institution add additional support.^20^ Argolo et al^20^ reported that trastuzumab and pertuzumab–based combinations in the second-line setting were associated with a median PFS of 10.3 months (95% CI, 5.9-16.3 months) vs 5.3 months (95% CI, 3.0-6.6 months) in patients treated with regimens that did not include pertuzumab (*P* = .03). In contrast, the PHEREXA trial^8^ demonstrated no significant PFS difference between capecitabine with trastuzumab plus pertuzumab vs capecitabine with trastuzumab alone for second-line treatment (9.0 vs 11.1 months; hazard ratio, 0.82; 95% CI, 0.65-1.02; *P* = .07). However, OS was 28.1 vs 36.1 months, favoring the pertuzumab-containing arm (HR, 0.68; 95% CI, 0.51-0.90). Although underpowered to evaluate OS, the signal of OS benefit in this study was consistent with the greater OS than PFS benefit with dual-antibody therapy in the CLEOPATRA study.^21^ Other trials are currently testing novel therapeutic strategies in the second-line setting. For example, in the phase 2 KATE2 trial,^22^ which evaluated the efficacy and safety of T-DM1 combined with atezolizumab, an anti–programmed death-ligand 1 antibody, vs T-DM1 combined with a placebo, there was no PFS benefit in the atezolizumab arm, and OS data are not yet mature. In addition to immunotherapy, other novel *ERBB2*-directed therapies include small-molecule tyrosine kinase inhibitors (eg, neratinib, afatinib, tucatinib, pyrotinib), antibody-drug conjugates, and bispecific antibodies. An important future direction will be to compare the efficacy of these emerging therapeutics with dual-antibody therapy (ie, pertuzumab plus trastuzumab) and with standard second-line T-DM1.

Our study demonstrated promising activity of dual-antibody therapy after prior exposure in the second-line setting and beyond. Notably, 49% of patients received T-DM1 prior to enrollment in this study. After exposure to pertuzumab and T-DM1, treatment practices vary and typically comprise chemotherapy combined with trastuzumab.^17^ In a small study of 29 patients, Cortés et al^9^ reported that the median PFS of dual-antibody therapy after disease progression on pertuzumab monotherapy was 4.4 months (80% CI, 1.5-7.3 months).^9^ In a heavily pretreated population of 602 patients enrolled in the TH3RESA trial,^10^ median PFS with treatment of physician’s choice (typically trastuzumab plus chemotherapy) was 3.3 months (95% CI, 2.9-4.1 months). Similarly, Blackwell et al^11^ reported a median PFS of 3 months in nearly 300 patients treated with trastuzumab and lapatinib after disease progression on trastuzumab-based regimens. Thus, in these 3 studies of more than 900 patients, the median PFS was approximately 3 to 4 months with standard options in the second-line setting and beyond. Although no direct cross-trial comparisons can be made, the median PFS of 5.5 months in the current study is encouraging.

Gemcitabine (at 1000 mg/m^2^) given with trastuzumab and pertuzumab was well tolerated. The most common grade 3 to 4 AEs were neutropenia and anemia, consistent with the known toxic profile of gemcitabine. Despite these toxic effects, median gemcitabine dose intensity was 1000 mg/m^2^, indicating that delivery of the full dose was feasible. Notably, the incidence of febrile neutropenia and grade 3 to 4 diarrhea were 0%. No patients experienced clinical heart failure (ie, symptomatic LVSD), and no patients withdrew from the study because of asymptomatic LVEF decline. One patient experienced asymptomatic LVEF decline, which recovered after antibody treatment was delayed, and treatment was resumed without further incident. Similar to trials in the first-line setting,^5,6^ the addition of pertuzumab did not increase cardiac toxic effects.

### Limitations and Strengths

This study has limitations. One limitation is its single-arm design; however, historical data were used to inform the efficacy target. Furthermore, this is a single-center study, and larger randomized clinical trials are needed. The study was strengthened by use of a single chemotherapeutic agent (ie, gemcitabine). Blood was collected throughout the study, and potential biomarkers of response will be reported separately. Additionally, an imaging substudy comparing response assessed by RECIST vs PERCIST criteria will be reported separately.

## Conclusions

This phase 2 clinical trial demonstrated promising efficacy and confirmed the tolerability and safety profile of gemcitabine with trastuzumab and pertuzumab for the treatment of *ERBB2*-positive metastatic breast cancer in the second-line setting and beyond. To our knowledge, this is the first study to show promising efficacy of a pertuzumab-containing regimen after prior exposure to pertuzumab. Our findings support the advancement of phase 3 trials comparing the efficacy of regimens containing trastuzumab and pertuzumab with standard and novel *ERBB2*-directed treatment in patients previously treated with this dual-antibody combination.

## Notes

Supplement 1. **Trial Protocol**

Supplement 2. **eFigure.** Study Flow Diagram
