# Supplementary material for: Efficacy and Safety of Gemcitabine With Trastuzumab and Pertuzumab After Prior Pertuzumab-Based Therapy Among Patients With Human Epidermal Growth Factor Receptor 2–Positive Metastatic Breast Cancer: A Phase 2 Clinical Trial
Source: JAMA Netw Open. 2019 Nov 27;2(11):e1916211. doi: 10.1001/jamanetworkopen.2019.16211 (PMC6902832; doi:10.1001/jamanetworkopen.2019.16211)
Supplement: Supplement 1. — Trial Protocol [file jamanetwopen-2-e1916211-s001.pdf]

Memorial Sloan Kettering Cancer Center
IRB Number: 14-124 A(15)
Approval Date: 01-DEC-2017

Phase II Study of Gemcitabine, Trastuzumab, and Pertuzumab in the Treatment of Metastatic HER2-Positive Breast Cancer after Prior Trastuzumab/Pertuzumab- or Pertuzumab-Based Therapy

PROTOCOL FACE PAGE FOR
MSKCC THERAPEUTIC/DIAGNOSTIC PROTOCOL

Principal Investigator/Department:	Chau Dang, MD	Medicine
Co-Principal Investigator(s)/Department:	Neil Iyengar, MD	Medicine

Investigator(s)/Department:	José Baselga, MD, PhD	Medicine
	Larry Norton, MD	Medicine
	Maura Dickler, MD	Medicine
	Sarat Chandarlapaty, MD	Medicine
	Shanu Modi, MD	Medicine
	Jacqueline Bromberg, MD, PhD	Medicine
	Teresa Gilewski, MD	Medicine
	Mark Robson, MD	Medicine
	Gabriella D'Andrea, MD	Medicine
	Diana Lake, MD	Medicine
	Andrew Seidman, MD	Medicine
	Nancy Sklarin, MD	Medicine
	Mary Ellen Moynahan, MD	Medicine
	Tiffany A. Traina, MD	Medicine
	Shari Goldfarb, MD	Medicine
	Victoria Blinder, MD	Medicine
	Monica Fornier, MD	Medicine
	Devika Gajria, MD, MPH	Medicine
	Elizabeth Comen, MD	Medicine
	Ayca Gucalp, MD	Medicine
	Komal Jhaveri, MD	Medicine
	Ting Bao, MD	Medicine
	Lillian Smyth, MD	Medicine
	Pedram Razavi, MD, PhD	Medicine
	Christopher Klebanoff, MD	Medicine
	Rui Wang, MD	Medicine
	Valentina Sterlin, NP	Nursing
	Lauren Frankel, RN	Nursing
	Stuart Lichtman, MD	Medicine
	Steven Sugarman, MD	Medicine
	Sarah Schweber, MD	Medicine
	Wanqing Iris Zhi, MD PhD	Medicine
	Roseann Caruso, NP	Nursing
	Leslie Matthews, NP	Nursing
	Lori Gofter, CRN	Nursing
	Audrey Hamilton, MD	Medicine
	Mila Gorsky, MD	Medicine
	Paul Hamlin, MD	Medicine
	Louise Ligresti, MD	Medicine
	Neha Korde, MD	Medicine
	Karen Dougherty, NP	Nursing
	Janet Cogswell, CRN	Nursing
	Kenneth Ng, MD	Medicine
	Zoe Goldberg, MD	Medicine
	TiffanyTroso-Sandoval, MD	Medicine
	Pamela Drullinsky, MD	Medicine

	Erin Scansorale, CRN	Nursing
	Krysti Corrado, CRN	Nursing
	Rachel Grisham, MD	Medicine
	Rachel Sanford, MD	Medicine
	Jasmeet Singh, MD	Medicine
	Karin Budrock, NP	Nursing
	Gloria Wasilewski, CRN	Nursing
	Jason Konner, MD	Medicine
	Serena Wong, MD	Medicine
	Colette Owens, MD	Medicine
	Loren Michel, MD	Medicine
	Marina Shcherba, DO	Medicine
	Azadeh Namakydoust, MD	Medicine
	Tina Alano, RN	Nursing
	Emer Heaney, RN	Nursing
	Maureen Cannon, NP	Nursing
	Karen Drucker, NP	Nursing
	Odessa Williams, NP	Nursing
	Danielle Alleva, NP	Nursing
	Nicole Devine, NP	Nursing
	Catherine Keane, NP	Nursing
	Renee Wisniewski, NP	Nursing
	Sujata Patil, PhD	Biostatistics
	Maxine Jochelson, MD	Radiology
	Gary Ulaner, MD, PhD	Radiology
	Sarah Hayes, MD	Radiology
	Lizza Lebron-Zapata,MD	Radiology
	Stefanie Jacobs, MD	Radiology

Consenting Professional(s)/Department:	Chau Dang, MD	Medicine
	Neil Iyengar, MD	Medicine
	Sarat Chandarlapaty, MD	Medicine
	Shanu Modi, MD	Medicine
	José Baselga, MD, PhD	Medicine
	Larry Norton, MD	Medicine
	Maura Dickler, MD	Medicine
	Jacqueline Bromberg, MD, PhD	Medicine
	Teresa Gilewski, MD	Medicine
	Mark Robson, MD	Medicine
	Gabriella D'Andrea, MD	Medicine
	Diana Lake, MD	Medicine
	Andrew Seidman, MD	Medicine
	Nancy Sklarin, MD	Medicine
	Mary Ellen Moynahan, MD	Medicine
	Tiffany A. Traina, MD	Medicine
	Shari Goldfarb, MD	Medicine
	Victoria Blinder, MD	Medicine
	Monica Fornier, MD	Medicine
	Devika Gajria, MD, MPH	Medicine
	Elizabeth Comen, MD	Medicine
	Ayca Gucalp, MD	Medicine
	Komal Jhaveri, MD	Medicine
	Ting Bao, MD	Medicine
	Stuart Lichtman, MD	Medicine
	Steven Sugarman, MD	Medicine
	Lillian Smyth, MD	Medicine
	Pedram Razavi, MD, PhD	Medicine
	Christopher Klebanoff, MD	Medicine
	Jasmeet Singh, MD Sarah Schweber, MD	Medicine
	Audrey Hamilton, MD	Medicine
	Mila Gorsky, MD	Medicine
	Paul Hamlin, MD	Medicine
	Louise Ligresti, MD	Medicine
	Pamela Drullinsky, MD	Medicine
	Zoe Goldberg, MD	Medicine
	Kenneth Ng, MD	Medicine
	TiffanyTroso-Sandoval, MD	Medicine
	Arlyn Apollo,MD	Medicine
	Neha Korde, MD	Medicine
	Rachel Grisham, MD	Medicine
	Rachel Sanford, MD	Medicine
	Wanqing Iris Zhi, MD PhD	Medicine
	Jason Konner, MD	Medicine
	Serena Wong, MD	Medicine
	Colette Owens, MD	Medicine
	Loren Michel, MD	Medicine
	Marina Shcherba, DO	Medicine
	Azadaeh Namakydoust, MD	Medicine
		Medicine

Please Note: A Consenting Professional must have completed the mandatory Human Subjects Education and Certification Program.

OneMSK Sites
Manhattan
Basking Ridge
Commack
Monmouth
Rockville Centre
Westchester

MSK Alliance Clinical Trials Sites	PI's Name	Site's Role
Hartford Healthcare	Patricia DeFusco, MD	Data Collection

Memorial Sloan-Kettering Cancer Center
1275 York Avenue
New York, New York 10065

Table of Contents

1.0 PROTOCOL SUMMARY AND/OR SCHEMA 6
2.0 OBJECTIVES AND SCIENTIFIC AIMS 6
3.0 BACKGROUND AND RATIONALE 7
4.0 OVERVIEW OF STUDY DESIGN/INTERVENTION 11
4.1 Design 11
4.2 Intervention 11
5.0 THERAPEUTIC/DIAGNOSTIC AGENTS 12
6.0 CRITERIA FOR SUBJECT ELIGIBILITY 18
6.1 Subject Inclusion Criteria 18
6.2 Subject Exclusion Criteria 19
7.0 RECRUITMENT PLAN 19
8.0 PRETREATMENT EVALUATION 19
9.0 TREATMENT/INTERVENTION PLAN 19
10.0 EVALUATION DURING TREATMENT/INTERVENTION 24
11.0 TOXICITIES/SIDE EFFECTS 26
12.0 CRITERIA FOR THERAPEUTIC RESPONSE/OUTCOME ASSESSMENT 30
13.0 CRITERIA FOR REMOVAL FROM STUDY 31
14.0 BIOSTATISTICS 31
15.0 RESEARCH PARTICIPANT REGISTRATION AND RANDOMIZATION PROCEDURES 33
15.1 Research Participant Registration 33
15.2 Randomization 33
16.0 DATA MANAGEMENT ISSUES 33
16.1 Quality Assurance 33
16.2 Data and Safety Monitoring 33
17.0 PROTECTION OF HUMAN SUBJECTS 34
17.1 Privacy 35
17.2 Serious Adverse Event (SAE) Reporting 36
17.2.1 Reporting SAEs 36
18.0 INFORMED CONSENT PROCEDURES 41
19.0 REFERENCES 42
20.0 APPENDICES 45

1.0 PROTOCOL SUMMARY AND/OR SCHEMA

Schema

Accrual Goal = 45 patients with MBC

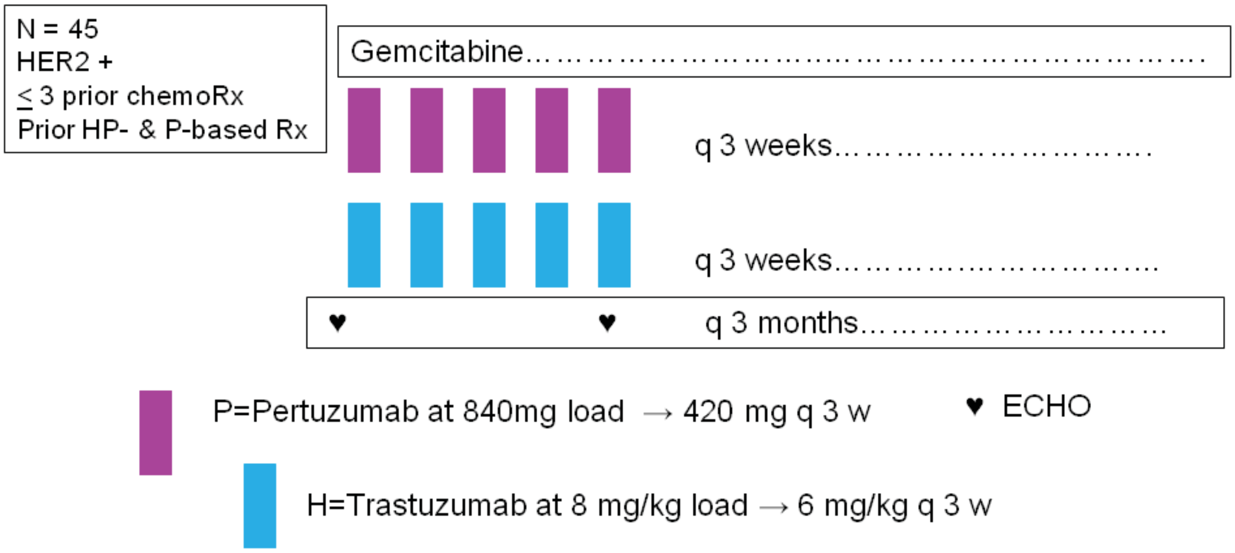

This is a phase II study of gemcitabine with trastuzumab and pertuzumab (HP) in patients with HER2 (+) metastatic breast cancer (MBC) who have had prior HP- or P-based treatment. Trastuzumab will be given IV every 3 weeks (8 mg/kg loading dose followed by 6 mg/kg every 3 weeks). Trastuzumab may be given IV weekly (4 mg/kg loading dose followed by 2 mg/kg weekly) in lieu of the every 3 week schedule, at the physician's discretion. Pertuzumab will be given IV at 840 mg as a loading dose followed by 420 mg every 3 weeks. Loading doses of trastuzumab and pertuzumab will not be required for patients who have received it < 6 weeks prior to Cycle 1 Day 1. We will assess for disease response at 12 weeks (+/- 7 days) from cycle 1 day 1 and then every 12 weeks (+/- 7 days) afterwards. We will also assess the left ventricular ejection fraction (LVEF) at baseline and at 12 weeks (+/- 7 days) from cycle 1 day 1 with an echocardiogram (ECHO). A multi-gated acquisition study may be ordered if an ECHO cannot be done. Exploratory studies will also be performed to compare response by PET response criteria (PRC) on 18-F FDG PET when compared with response by RECIST on dedicated CT.

2.0 OBJECTIVES AND SCIENTIFIC AIMS

- The primary objective of this study will be to assess 3-month PFS under treatment with gemcitabine + HP in patients with HER2 (+) MBC with prior HP- or P-based treatment but no more than 3 prior chemotherapy regimens for MBC.
- The secondary objectives will be to assess progression-free survival, response, overall survival, and safety (including cardiac safety) and tolerability. A cardiac event will be defined as a) "symptomatic" left ventricular systolic dysfunction (LVSD) (deaths and non-deaths), b) non-LVSD cardiac death, or c) probable cardiac death.
- An exploratory endpoint will be to compare response by PRC on 18-F FDG-PET with CT by RECIST on dedicated CT (Hypothesis is that that physiologic imaging with 18-F FDG PET/CT is superior to CT scans in predicting progression free survival).

3.0 BACKGROUND AND RATIONALE

Despite significant advances in the past decade, breast cancer remains a leading cause of death and the most common cancer in women in the U.S. Approximately 200,000 new cases are diagnosed in the U.S. each year with more than 1 million new cases diagnosed worldwide.[1] More recent therapeutic innovations have been developed largely in part due to improving recognition of biologically distinct breast cancer subsets. These subsets are currently defined by hormone receptor and HER2 status, which allow treatment recommendations to be tailored to these features.

The human epidermal growth factor receptor 2 (HER2/*neu*) is a proto-oncogene that encodes the 185kDa HER2 protein, a transmembrane tyrosine kinase receptor that is a member of the human epidermal growth factor receptor family. HER2, which is involved in the regulation of cell growth and survival, is amplified in 15-20% of invasive breast carcinomas and confers poor prognosis with increased risk of disease progression and decreased OS.[2] Trastuzumab (Herceptin®)(H) is a humanized monoclonal antibody that binds to the extracellular domain of HER2 and thereby limits its activation. While the exact mechanism of action of trastuzumab in vivo is not fully understood, it is known to exert many antitumor effects including disruption of intracellular signaling leading to cell cycle arrest and apoptosis,[3,4] antibody-dependent cellular toxicity,[5] and inhibition of angiogenesis.[6] In clinical trials, trastuzumab has improved outcomes for patients with both metastatic and early stage HER2 positive breast cancer.[7-9] Trastuzumab is active as a single agent and acts synergistically with several chemotherapy agents with response rates of 35% in the first-line setting and 11.6%-15% in pretreated patients.[7,10-14] The addition of trastuzumab to chemotherapy for the treatment of HER2-overexpressing metastatic breast cancer has improved time to progression (TTP), time to treatment failure (TTF), overall response rate (ORR), duration of response (DOR), and overall survival (OS) when compared to chemotherapy alone in the front-line setting.[7,10]

In the pivotal phase III first-line trial in which patients were randomized to an anthracycline or

paclitaxel with or without trastuzumab, Slamon et al reported improvements in TTP (7.4 vs. 4.6 months $p<0.001$), DOR (9.1 vs. 6.1 months, $p<0.001$), median TTF (6.9 vs. 4.5 months, $p<0.001$), and median OS (25.1 vs. 20.3 months, $p<0.001$) in the trastuzumab vs. chemotherapy alone arms.[7] Similar results were reported by Marty and colleagues in a randomized trial of trastuzumab plus docetaxel vs. docetaxel alone.[10] In this trial, 186 patients were randomized to 6 cycles of docetaxel 100 mg/m^2 every 3 weeks with or without trastuzumab until disease progression. Significant improvements in ORR (61% vs. 34%, $p=0.0002$), median TTF (9.8 vs. 5.3 months, $p=0.0001$), median TTP (11.7 vs. 6.1 months, $p=0.0001$), median DOR (11.7 vs. 5.7 months, $p=0.009$), and median OS (31.2 vs. 22.7 months $p=0.0325$) were observed in the trastuzumab containing arm.

Other front-line trials have shown response rates of 62%-84% when trastuzumab is combined with vinorelbine,[15,16] and 63%-65% when combined with capecitabine.[17] Beyond the first-line setting, the combination of trastuzumab with chemotherapy including paclitaxel, docetaxel, vinorelbine, gemcitabine, or capecitabine produces response rates ranging from 20%-55%.[17-24]

However, despite these therapeutic improvements, the large majority of patients will develop progressive disease and ultimately succumb to metastatic breast cancer.[1] This has spurred the development of newer HER2 directed therapies.

Pertuzumab

Pertuzumab (P) is a monoclonal antibody that binds to the extracellular domain of HER2. However, unlike trastuzumab, which binds at domain IV, P binds domain II and is thus able to disrupt HER2 dimerization and ligand-activated signaling with other growth factor receptors.[25] Preclinical data have shown synergistic activity of HP.[26-28] Clinical activity has been observed in patients with HER2 low-expressing tumors who have received P.[29] Portera et al conducted a study that evaluated the efficacy of HP in 11 patients with HER2 (+) breast cancer (BCA). A clinical benefit rate (CBR) of 27% was demonstrated.[30] Baselga et al reported the result of the phase II study of HP in 66 patients who had progression after H-based therapy (Rx) and showed that the overall response rate (ORR) and CBR were 24% and 50%, respectively.[31] The promising results of the dual-anti-HER2 antibody Rx led to the inclusion of a third cohort of patients receiving P alone. Upon progression on P, H can be added to P at the physician's discretion. Cortes et al. reported the result of P and showed that the ORR and CBR were 3.4% and 10.3%, respectively.[32] These rates were improved when H was added back, rendering an ORR and CBR of 17.6% and 41.2%, respectively. The median progression-free survival (PFS) was 7.1 weeks vs 17.4 weeks, for P vs HP, respectively. Thus, it is clear that dual anti-HER2 antibody Rx was better than P alone.

The results of these trials led to the conduct of the randomized phase III CLEOPATRA study

led by Baselga et al.[33] This study randomized 808 patients with HER2 (+) metastatic breast cancer (MBC) to docetaxel/H + placebo/ P in the first-line setting. Overall, the median PFS was in favor of the HP containing arm (12.4 vs. 18.5 months, p < 0.001). Recently Swain et al showed that there was statistically significant gain in overall survival (OS) in favor of the HP-containing arm (HR 0.66, p = 0.0008).[34] This result has changed the paradigm of treatment for patients with HER2 (+) MBC in the first-line setting. Additionally, preliminary data from a small phase II study of weekly paclitaxel + HP showed that this combination is also very active, as measured by PFS and response rate.[35] The National Comprehensive Cancer Network (NCCN) guidelines have endorsed a combination of taxane/HP as first-line Rx for HER2 (+) MBC based on these studies.[33-35]

Antibody Drug Conjugates

Ado-Trastuzumab emtansine (TDM-1) is an antibody-drug conjugate that combines trastuzumab with the potent antimicrotubule agent, DM1. This combination allows for intracellular drug delivery of the cytotoxic DM1 with trastuzumab specifically to HER2-over-expressing cells and therefore minimizes exposure of normal cells while enhancing therapeutic index. Two phase II trials of TDM-1 showed promising activity of TDM-1 in previously treated patients.[42,43] Hurvitz et al presented results of a randomized phase II study comparing docetaxel/H to TDM-1 in 137 patients with HER2(+) MBC in the first-line setting. Median PFS was in favor of the TDM-1 arm (14.2 vs. 9.2 months, p = 0.035).[44] Regarding the definitive activity of TDM-1 in the first-line setting, the MARIANNE study was a randomized phase III study comparing a taxane plus trastuzumab to TDM-1 alone and to TDM-1 plus pertuzumab. Recently, these results were reported at ASCO 2015 demonstrating that TDM-1 and TDM-1/P were not superior to standard taxane/H.[52] Thus, TDM-1 does not replace the current first-line standard treatment with taxane/HP. However, TDM-1 does have a role in the second line setting. In the 2nd-line setting, the phase III study (EMILIA) compared TDM-1 to capecitabine/lapatinib for 991 patients. The study showed that TDM-1 was superior in terms of median PFS (9.6 vs. 6.4 months, p < 0.001) and OS (30.9 vs. 25.1 months, p < 0.001).[39] Based on the EMILIA data, TDM-1 is approved for the treatment of patients with HER2 (+) MBC who have had prior treatment with a taxane and H. In the 3rd-line setting, Wildiers et al recently reported the results of the TH3RESA study in 600 patients with ≥ 2 prior HER2-directed therapy who were previously treated with a taxane, trastuzumab, and lapatinib were randomized to TDM-1 versus treatment of physician's choice (TPC). In this study, 80.4% of the patients had prior trastuzumab-based therapy (68.5% chemotherapy + H, 10.3% H + lapatinib, and 1.6% H + hormonal therapy). Overall, TDM-1 was superior to TPC arm with median PFS of 6.2 vs 3.3 months (p< 0.0001). The OS data were immature but did show a trend favoring TDM-1.[40] The median PFS of 3.3 months in the TPC arm was similar to the median PFS of 12 weeks (3 months) seen with

dual anti-HER2 therapy with H + lapatinib (L) versus lapatinib alone (8 weeks) in another randomized study of 296 patients who were heavily pretreated, reported by Blackwell et al. 41

The NCCN endorsed several options in the 2nd-line setting and beyond and they included not only TDM-1 but also, capecitabine + L, H + any chemotherapy or hormonal therapy, HL, HP +/- cytoxic therapy. [42]

Rationale:

With the FDA approval and NCCN endorsement of taxane/HP in the first-line setting in metastatic disease, most patients will have received HP-based therapy. Some may have received pertuzumab with TDM-1 in the metastatic trial (i.e. MARIANNE) or neoadjuvant or adjuvant trials. Thus, we will enroll patients who had exposure to HP- or P-based therapy in neoadjuvant, adjuvant, or metastatic setting. It is anticipated that most patients enrolled onto this study will be those who had prior exposure to HP- or P-based therapy in the metastatic setting. Although taxane/HP has excellent activity in the metastatic setting, it is inevitable that most patients' tumors will become resistant to these treatments. This will be the first trial evaluating the efficacy of dual anti-HER2 antibody (HP) with chemotherapy beyond HP- or P-based therapy. Cortes et al demonstrated that the median PFS of HP (after H-based treatment and P monotherapy) was 4.35 months in a study of 29 patients.[32] In a heavily pretreated population of 600 patients, Wildiers et al reported a median PFS of 3.3 months with TPC (mainly standard therapy of H + other agents) in a randomized study. [40] In another study of a heavily pretreated population of 296 patients, Blackwell et al reported that the median PFS was 3 months with a standard arm of HL.[41] Thus, in these 3 studies of over 900 patients, the median PFS was in the order of 3-4 months with standard options in the 2nd-line setting and beyond. The hypothesis of this trial is that HP + gemcitabine after HP-or P-based therapy would be better than the median PFS of 3-4 months reported in the historical control. The proposed trial is important as it will determine the efficacy of HP-based treatment after HP- or P-based treatment. Based on historical controls, we will use the median PFS of 3 months as the benchmark. We hypothesize that in this study of gemcitabine/HP, the median PFS will be better than 3 months and that the median 3-month PFS with gemcitabine/HP in our study will be greater than 50%. Eligible patients will be those with measurable or non-measurable HER2 (+) MBC who have had prior HP- or P-based treatment but no more than 3 prior lines of chemotherapy-based treatment for HER2 (+) MBC.

Cardiac Safety:

In the seminal trastuzumab trial reported by Slamon et al., cardiac toxicity with trastuzumab was accentuated in combination with anthracycline if given at a cumulative dose greater than 300 mg/m^2 (27% incidence with anthracycline and trastuzumab, 13% with paclitaxel and

trastuzumab, and 8% with anthracycline therapy alone).[7] Pooled analysis for trastuzumab-related cardiac toxicity from seven phase II and III clinical trials have been reported.[43-44] Independent analysis by the Cardiac Review and Evaluation Committee evaluated risk and severity of cardiac dysfunction, baseline risk factors, and the role of cumulative doses of anthracyclines and trastuzumab relative to the development of cardiac dysfunction.
The New York Heart Association (NYHA) functional classification system was used to categorize the severity of these events. The incidence of class III or IV cardiac dysfunction was highest,
16%, for patients who received concurrent AC or EC and trastuzumab (again, these toxicities were noted for patients receiving the higher dose levels of anthracyclines). Whereas the incidence was 1% in patients receiving paclitaxel alone, 2% for those who received first-line trastuzumab, 4% for those who received trastuzumab in the refractory setting, and 2% for those receiving concurrent paclitaxel plus trastuzumab.

The cardiac toxicity profile of pertuzumab has been low.[45] Our previous study (IRB # 10-142) with paclitaxel, trastuzumab, and pertuzumab demonstrated that no patients experienced clinical heart failure. [35] From the CLEOPATRA study and our study (IRB #10-142), pertuzumab with trastuzumab is associated with only rare rates of grade 3-4 left ventricular systolic dysfunction (LVSD) (symptomatic heart failure) in the order of 0-1%.

<u>PET Response Criteria (PRC)</u>

RECIST criteria have been the standard method for assessing response to chemotherapy, particularly in patients with solid tumors. However there are significant limitations to RECIST which can impair the ability to evaluate new chemotherapy protocols. Lesions such as bone metastases, inflammatory breast cancer, leptomeningeal disease, and effusions which are frequently seen in breast cancer are non measurable and difficult to even evaluate for response using CT. Evaluation of anatomic change using RECIST does not correlate with pathologic response as well as morphologic and metabolic criteria particularly in patients receiving targeted therapy (36, 37,38) The Cheson Criteria using changes in both anatomic measurement and FDG activity is a far more accurate method of evaluating response to treatment and has largely replaced RECIST in patients with lymphoma. As a result risk adaptive treatments are being widely explored.

It has been shown in multiple studies that PET/CT can accurately predict response to treatment for breast cancer after one or 2 cycles of chemotherapy (39,40,41). This would be extremely useful for the purposes of this study for which early time points in evaluating response will be of value. Since both PET and diagnostic CT will be performed, we will be able to compare the ability of the 2 tests to predict progression free survival.

4.0 OVERVIEW OF STUDY DESIGN/INTERVENTION

4.1 Design

This is a phase II study of gemcitabine with trastuzumab and pertuzumab (HP) in patients with HER2 (+) metastatic breast cancer (MBC) who have had prior HP- and P-based treatment. Trastuzumab will be given IV every 3 weeks (8 mg/kg loading dose followed by 6 mg/kg every 3 weeks). A loading dose of trastuzumab will not be required for patients who have received it < 6 weeks prior to Cycle 1 Day 1.

Trastuzumab may be given IV weekly (4 mg/kg loading dose followed by 2 mg/kg weekly) in lieu of the every 3 week schedule, at the physician's discretion. Pertuzumab will be given IV at 840 mg as a loading dose followed by 420 mg every 3 weeks. A loading dose of pertuzumab will not be required for patients who have received it < 6 weeks prior to Cycle 1 Day 1. We will assess for disease response every 12 weeks (+/- 7 days) from cycle 1 day 1. We will also assess the left ventricular ejection fraction (LVEF) at baseline and at 12 weeks (+/- 7 days) with an echocardiogram (ECHO). A multi-gated acquisition study may be ordered if an ECHO cannot be done. After the 12 week time point from cycle 1 day 1, subsequent LVEF assessment may be done infrequently and at the discretion of the treating physician. This is based on a large body of evidence and from our study (IRB #10-142) that pertuzumab with trastuzumab is associated with only rare rates of grade 3-4 LVSD (symptomatic heart failure) in the order of 0-1%.

4.2 Intervention

Given the success of taxane/HP in the first-line setting, most patients will have been exposed to taxane/HP in the first or second-line setting. Some may have received pertuzumab with TDM-1 in the metastatic trial (i.e. MARIANNE) or neoadjuvant or adjuvant trials. Thus, we will enroll patients who had exposure to HP- or P-based therapy in the neoadjuvant, adjuvant, or metastatic settings. This will be the first trial to evaluate the efficacy of dual anti-HER2 antibody (HP) with gemcitabine beyond HP- or P-based treatment. The regimen will consist of gemcitabine at 1000 mg/m^2 IV weekly days 1 + 8 q 3 weeks + trastuzumab every 3 weeks (8 mg/kg loading dose followed by 6 mg/kg every 3 weeks) + pertuzumab every 3 weeks (840 mg as a loading dose followed by 420 mg every 3 weeks), all given intravenously (IV). Trastuzumab may be given IV weekly (4 mg/kg loading dose followed by 2 mg/kg weekly) in lieu of the every 3 week schedule. Loading doses of trastuzumab and pertuzumab will not be required for patients who have received it < 6 weeks prior to Cycle 1 Day 1.

The primary endpoint of this study will be the proportion of patients who are progression free at 3 months or later. Patients who are considered progression-free at 3 months are deemed successes. Treatment failures are those patients whose disease progressed before the 3 month mark. The secondary endpoints will be response, overall survival, and safety (including

cardiac safety) and tolerability. We will also assess the LVEF at baseline and at 12 weeks (+/- 7 days) from cycle 1 day 1 with an ECHO. When an ECHO cannot be done, a MUGA scan may be done. After the 12 week time point from cycle 1 day 1, subsequent LVEF assessment will be at the discretion of the treating physician. This is based on a large body of evidence and from our study (IRB #10-142) that pertuzumab with trastuzumab is associated with only rare rates of grade 3-4 LVSD (symptomatic heart failure) in the order of 0-1%.

5.0 THERAPEUTIC/DIAGNOSTIC AGENTS

Trastuzumab (Herceptin®)

a. Dosage

The recommended initial loading Herceptin dose is 4 mg/kg (for weekly dosing schedules) or 8 mg/kg (for every 3 weeks) administered over approximately 90 minutes. A loading dose of trastuzumab will not be required for patients who have received it < 6 weeks prior to Cycle 1 Day 1. The recommended maintenance Herceptin dose is 2 mg/kg weekly or 6 mg/kg every 3 weeks and can be administered over approximately 30 minutes if the initial loading dose was well tolerated. Herceptin may be administered in an outpatient setting. DO NOT ADMINISTER AS AN IV PUSH OR BOLUS (see ADMINISTRATION).

b. Preparation

Use appropriate aseptic technique. Each vial of Herceptin should be reconstituted with 20 mL of BWFI, USP, 1.1% benzyl alcohol preserved, as supplied, to yield a multidose solution containing 21 mg/mL Herceptin. Immediately upon reconstitution with BWFI, the vial of Herceptin must be labeled in the area marked "Do not use after" with the future date that is 28 days from the date of reconstitution.

If the patient has known hypersensitivity to benzyl alcohol, Herceptin must be reconstituted with Sterile Water for Injection (see PRECAUTIONS). Herceptin which has been reconstituted with SWFI must be used immediately and any unused portion discarded. Use of other reconstitution diluents should be avoided.

Determine the dose of Herceptin needed. Calculate the correct dose using 21 mg/mL Herceptin solution. Withdraw this amount from the vial and add it to an infusion bag containing 250 mL of 0.9% sodium chloride, USP. DEXTROSE (5%) SOLUTION SHOULD NOT BE USED. Gently invert the bag to mix the solution. The reconstituted preparation results in a colorless to pale yellow transparent solution. Parenteral drug products should be inspected visually for particulates and discoloration prior to administration. No incompatibilities between Herceptin and polyvinylchloride or polyethylene bags have been observed.

c. Administration

Treatment may be administered in an outpatient setting by administration of a 4 mg/kg Herceptin loading dose for weekly dosing schedules (OR 8 mg/kg Herceptin loading dose for q3wk dosing schedules) by intravenous (IV) infusion given over approximately 90 minutes. DO NOT ADMINISTER AS AN IV PUSH OR BOLUS. If Herceptin is being administered concomitantly with chemotherapy, Herceptin administration may be given before or after

chemotherapy administration. Patients should be observed for fever and chills or other infusion-associated symptoms (see ADVERSE REACTIONS). If prior infusions are well tolerated subsequent doses of 2 mg/kg Herceptin weekly (OR 6 mg/kg Herceptin q3wk) may be administered over approximately 30 minutes.

Herceptin should not be mixed or diluted with other drugs. Herceptin infusions should not be administered or mixed with Dextrose solutions.

d. Storage

Vials of Herceptin are stable at 2°C–8°C (36°F–46°F) prior to reconstitution. Do not use beyond the expiration date stamped on the vial. A vial of Herceptin reconstituted with BWFI, as supplied, is stable for 28 days after reconstitution when stored refrigerated at 2°C–8°C (36°F–46°F), and the solution is preserved for multiple use. Discard any remaining multi-dose reconstituted solution after 28 days. If unpreserved SWFI (not supplied) is used, the reconstituted Herceptin solution should be used immediately and any unused portion must be discarded. DO NOT FREEZE HERCEPTIN THAT HAS BEEN RECONSTITUTED.

The solution of Herceptin for infusion diluted in polyvinylchloride or polyethylene bags containing 0.9% sodium chloride for injection, USP, may be stored at 2°C–8°C (36°F–46°F) for up to 24 hours prior to use. Diluted Herceptin has been shown to be stable for up to 24 hours at room temperature 15°C–25°C; however, since diluted Herceptin contains no effective preservative the reconstituted and diluted solution should be stored refrigerated (2°C–8°C).

e. Safety

Infusion-Associated Symptoms. During the first infusion with Herceptin, a symptom complex consisting of chills and/or fever is observed in approximately 40% of patients. Other signs and/or symptoms may include nausea, vomiting, pain, rigors, headache, cough, dizziness, rash, and asthenia. These symptoms are usually mild to moderate in severity, and occur infrequently with subsequent Herceptin infusions. These symptoms can be treated with an analgesic/antipyretic such as meperidine or paracetamol, or an antihistamine such as diphenhydramine.

Serious Infusion-Associated Events. Serious adverse reactions to Herceptin infusion including dyspnea, hypotension, wheezing, bronchospasm, tachycardia, reduced oxygen saturation and respiratory distress have been reported infrequently. In rare cases (4 per 10,000), these events were associated with a clinical course culminating in a fatal outcome. Serious reactions have been treated with supportive therapy such as oxygen, beta-agonists, corticosteroids and withdrawal of Herceptin as indicated.

Hematologic Toxicity. In the clinical trials, an increased incidence of anemia was observed in patients receiving Herceptin plus chemotherapy compared with patients receiving chemotherapy alone. The majority of these anemia events were mild or

Pertuzumab (Perjeta)

a. Dosage

Pertuzumab is a humanized monoclonal antibody based on the human IgG1 framework sequences and consists of 2 heavy chains and 2 light chains. Pertuzumab will be administered as 840 mg loading dose followed by 420 mg IV every 3 weeks. A loading dose of pertuzumab will not be required for patients who have received it < 6 weeks prior to Cycle 1 Day 1.

b. Preparation

Pertuzumab is produced in Chinese hamster ovary cell cultures and purified by protein A column affinity chromatography, followed by ion exchange column chromatography. Because of the high degree of homology between pertuzumab and trastuzumab, procedures similar to those developed for trastuzumab are used for the manufacturing process, the in-process controls, and the characterization of pertuzumab. No bovine-derived raw materials are used in the manufacture of pertuzumab.

Each lot of the recombinant antibody produced for clinical purposes meets the USP requirements for sterility and safety. Additionally, each lot is extensively characterized and meets the required specifications for identity, purity, and potency.

Pertuzumab is provided as a single use formulation containing 30 mg/ml in 20 mM L-histidine acetate (pH 6.0), 120 nM sucrose and 0.02% polysorbate 20. Each 20 ml vial contains 420 mg of pertuzumab (14.0 ml/vial).

c. Administration

In the clinical trials, the per-patient incidences of moderate to severe neutropenia and of febrile neutropenia were higher in patients receiving Perjeta in combination with myelosuppressive chemotherapy as compared to those who received chemotherapy alone.

In the post marketing setting, deaths due to sepsis in patients with severe neutropenia have been reported in patients receiving Perjeta and myelosuppressive chemotherapy, although in controlled clinical trials (pre- and post-marketing), the incidence of septic deaths was not significantly increased. The pathophysiologic basis for exacerbation of neutropenia has not been determined; the effect of Perjeta on the pharmacokinetics of chemotherapeutic agents has not been fully evaluated. The observed incidence of leukemia among Perjeta-treated patients appears to be consistent with the expected incidence of leukemia among patients treated with chemotherapy for metastatic breast cancer. Therefore, the contribution of Herceptin to the etiology of acute leukemia or myelodysplastic syndrome in these cases is unclear.

Treatment may be administered in an outpatient setting by administration of 840 mg followed by 420 mg IV every 3 weeks. The first two doses will be given over approximately 60 minutes and the subsequent doses will be given over approximately 30-60 minutes. If pertuzumab is being administered concomitantly with trastuzumab and paclitaxel, there is no strict sequence of administration of these 3 drugs mandated. Patients should be observed for fever and chills

or other infusion- associated symptoms.

d. Storage

Pertuzumab vials are to be refrigerated at 2º C-8º C (36º F-46º F) until use. Pertuzumab vials should not be used beyond the expiration date provided by the manufacturer (Genentech). Because the formulation does not contain a preservative, the vial seal may only be punctured once. Any remaining solution should be discarded. Vial contents should not be frozen. The solution of pertuzumab fro infusion diluted in PVC or non-PVC polyolefin bags containing 0.9% sodium chloride injection and may be stored at 2º C-8º C (36º F-46º F) for up to 24 hours prior to use. Diluted pertuzumab has been shown to be stable for up to 24 hours at room temperature (2º C-25º C). However, since diluted pertuzumab contains no preservative, the diluted solution should be stored refrigerated at 2º C-8º C.

e. Safety

As of November of 2008, approximately 840 patients with advanced cancers or early stage HER2 (+) breast cancer have been treated with pertuzumab. Gastrointestinal toxicities (diarrhea, nausea, vomiting, abdominal pain) and fatigue are the most frequently reported adverse events (AEs) with single therapy.

Diarrhea and rash

Although pertuzumab targets HER2, because of its role in heterodimerization with other members of the HER family (i.e., HER1/EGFR), it may cause toxicities associated with the use of EGFR inhibitors. Diarrhea and rash are common events increased with pertuzumab given in combination with chemotherapy compared with chemotherapy alone. Diarrhea has been reported in 60-70% of patients treated with pertuzumab and was mostly of grades 1-2.
The mechanism of diarrhea and rash are unknown, but the nature is similar to that of other agents causing HER1 inhibition. In the event of diarrhea, early intervention with anti-diarrhea medication should be considered and patients treated with fluid and electrolyte replacement, as clinically indicated.

Infusion-related Symptoms

Monoclonal antibodies may cause infusion-associated symptoms such as fever, chills, hypotension, shortness of breath, skin rash, headache, nausea, and/or vomiting. Serious or severe infusion-related symptoms have been rarely observed with 7 patients experiencing serious reactions to date [hypersensitivity, fatal ARDS, pulmonary edema, anaphylaxis, dyspnea with hypertension, infusion-related reaction (unsteady on feet, headache, blurred vision)]. In pertuzumab single-agent studies, less than 5% of patients experienced adverse reactions during pertuzumab infusions. Serious infusion-related reactions with pertuzumab have been infrequently reported (< 1%). Intravenous pertuzumab administration should be performed in the setting with emergency equipment and staff who re trained to monitor medical

situations and respond to medical emergencies. Patients should be monitored during each pertuzumab infusion for any adverse effects, as infusion reactions may occur with the first or subsequent doses. The infusion should be stopped for patients who develop dyspnea, clinically significant hypotension, or other clinically significant events. Patients who experience an NCI-CTC grade 4 allergic reaction or acute respiratory distress syndrome should not receive additional pertuzumab.

Cardiac Toxicities

Since pertuzumab targets HER2, as with trastuzumab, there is a potential risk of cardiac dysfunction, particularly in patients who have received prior anthracycline treatment. Cardiac toxicities, predominantly asymptomatic left ventricular ejection fraction (LVEF) declines, and 4 cases of cardiac failure have been reported in approximately 840 patients with advanced malignant disease or early stage HER2 (+) breast cancer treated with pertuzumab. Two of these cases occurred in patients with metastatic breast cancer who had received prior anthracyclines and two in patients with ovarian cancer. No clear association between the frequency, nature, and severity of pertuzumab-related toxicities and dose level has been observed.

Patients with significant cardiac disease or baseline LVEF below the institution’s lower limit of normal (LLN) should not commence treatment with pertuzumab. Risk factors for pertuzumab-associated cardiac dysfunction are not known at this time. This risk should be carefully weighed against the potential benefit in patients who have received prior anthracyclines. Monitoring of the LVEF is advised while patients are receiving pertuzumab. If symptomatic LVEF decline develops (NCI-CTC grade 3 or 4), the patient must discontinue pertuzumab. Left ventricular dysfunction, symptomatic or not, should be treated and followed according to standard medical practice.

Gemcitabine (Gemzar)

a. Dosage

The recommended dose of gemcitabine is 800-1250 mg/m^2 given IV weekly on days 1 and 8 every 21 days.

b. Preparation

Caution should be exercised in handling and preparing Gemzar solutions. The use of gloves is recommended. If Gemzar solution contacts the skin or mucosa, immediately wash the skin thoroughly with soap and water or rinse the mucosa with copious amounts of water. Although acute dermal irritation has not been observed in animal studies, 2 of 3 rabbits exhibited drug-related systemic toxicities (death, hypoactivity, nasal discharge, shallow breathing) due to dermal absorption.

c. Administration

The recommended diluent for reconstitution of Gemzar is 0.9% Sodium Chloride Injection

without preservatives. Due to solubility considerations, the maximum concentration for Gemzar upon reconstitution is 40 mg/mL. Reconstitution at concentrations greater than 40 mg/mL may result in incomplete dissolution, and should be avoided. To reconstitute, add 5 mL of 0.9% Sodium Chloride Injection to the 200-mg vial or 25 mL of 0.9% Sodium Chloride Injection to the 1-g vial. Shake to dissolve. These dilutions each yield a gemcitabine concentration of 38 mg/mL which includes accounting for the displacement volume of the lyophilized powder (0.26 mL for the 200-mg vial or 1.3 mL for the 1-g vial). The total volume upon reconstitution will be 5.26 mL or 26.3 mL, respectively. Complete withdrawal of the vial contents will provide 200 mg or 1 g of gemcitabine, respectively. Prior to administration the appropriate amount of drug must be diluted with 0.9% Sodium Chloride Injection. Final concentrations may be as low as 0.1 mg/mL. Reconstituted Gemzar is a clear, colorless to light straw-colored solution. After reconstitution with 0.9% Sodium Chloride Injection, the pH of the resulting solution lies in the range of 2.7 to 3.3. The solution should be inspected visually for particulate matter and discoloration prior to administration, whenever solution or container permit. If particulate matter or discoloration is found, do not administer.
When prepared as directed, Gemzar solutions are stable for 24 hours at controlled room temperature 20° to 25°C (68° to 77°F) [see USP Controlled Room Temperature]. Discard unused portion. Solutions of reconstituted Gemzar should not be refrigerated, as crystallization may occur.

d. Safety

Hematologic — In studies <1% of patients discontinued therapy for either anemia, leukopenia, or thrombocytopenia. Red blood cell transfusions were required by 19% of patients. The incidence of sepsis was less than 1%. Petechiae or mild blood loss (hemorrhage), from any cause, was reported in 16% of patients; less than 1% of patients required platelet transfusions. Patients should be monitored for myelosuppression during Gemzar therapy and dosage modified or suspended according to the degree of hematologic toxicity.

Gastrointestinal — Nausea and vomiting were commonly reported (69%) but were usually of mild to moderate severity. Severe nausea and vomiting (WHO Grade 3/4) occurred in <15% of patients. Diarrhea was reported by 19% of patients, and stomatitis by 11% of patients.

Hepatic — In clinical trials, Gemzar was associated with transient elevations of one or both serum transaminases in approximately 70% of patients, but there was no evidence of increasing hepatic toxicity with either longer duration of exposure to Gemzar or with greater total cumulative dose. Serious hepatotoxicity, including liver failure and death, has been reported very rarely in patients receiving Gemzar alone or in combination with other potentially hepatotoxic drugs.

Renal — In clinical trials, mild proteinuria and hematuria were commonly reported. Clinical findings consistent with the Hemolytic Uremic Syndrome (HUS) were reported in 6 of 2429 patients (0.25%) receiving Gemzar in clinical trials. Four patients developed HUS on Gemzar therapy, 2 immediately posttherapy. The diagnosis of HUS should be considered if the patient develops anemia with evidence of microangiopathic hemolysis, elevation of bilirubin or LDH, reticulocytosis, severe thrombocytopenia, and/or evidence of renal failure (elevation of serum creatinine or BUN). Gemzar therapy should be discontinued immediately. Renal failure may not be reversible even with discontinuation of therapy and dialysis may be required.

Fever — The overall incidence of fever was 41%. This is in contrast to the incidence of infection (16%) and indicates that Gemzar may cause fever in the absence of clinical infection. Fever was frequently associated with other flu-like symptoms and was usually mild and clinically manageable.

Rash — Rash was reported in 30% of patients. The rash was typically a macular or finely granular maculopapular pruritic eruption of mild to moderate severity involving the trunk and extremities. Pruritus was reported for 13% of patients.

Pulmonary — In clinical trials, dyspnea, unrelated to underlying disease, has been reported in association with Gemzar therapy. Dyspnea was occasionally accompanied by bronchospasm. Pulmonary toxicity has been reported with the use of Gemzar. The etiology of these effects is unknown. If such effects develop, Gemzar should be discontinued. Early use of supportive care measures may help ameliorate these conditions.

Edema — Edema (13%), peripheral edema (20%), and generalized edema (<1%) were reported. Less than 1% of patients discontinued due to edema.

Flu-like Symptoms — Flu syndrome< was reported for 19% of patients. Individual symptoms of fever, asthenia, anorexia, headache, cough, chills, and myalgia were commonly reported. Fever and asthenia were also reported frequently as isolated symptoms. Insomnia, rhinitis, sweating, and malaise were reported infrequently. Less than 1% of patients discontinued due to flu-like symptoms.

Infection — Infections were reported for 16% of patients. Sepsis was rarely reported (<1%).

Alopecia — Hair loss, usually minimal, was reported by 15% of patients.
Neurotoxicity — There was a 10% incidence of mild paresthesias and a <1% rate of severe paresthesias.

Extravasation — Injection-site related events were reported for 4% of patients. There were no reports of injection site necrosis. Gemzar is not a vesicant.

Allergic — Bronchospasm was reported for less than 2% of patients. Anaphylactoid reaction has been reported rarely. Gemzar should not be administered to patients with a known hypersensitivity to this drug.

Cardiovascular — During clinical trials, 2% of patients discontinued therapy with Gemzar due to cardiovascular events such as myocardial infarction, cerebrovascular accident, arrhythmia, and hypertension. Many of these patients had a prior history of cardiovascular disease.

6.0 CRITERIA FOR SUBJECT ELIGIBILITY

6.1 Subject Inclusion Criteria

- Age ≥ 18

- Stage IV HER2 (+) breast cancer
- Histologically documented HER2 (+) breast cancer as defined as IHC 3+ or FISH amplification of > 2.0 of primary or metastatic site; results from the local lab are acceptable.
- ECOG performance status 0 -1
- Prior treatment with trastuzumab + pertuzumab (HP)-based or pertuzumab-based therapy in the neoadjuvant/adjuvant, unresectable, locally advanced, or metastatic setting.
- < 3 prior chemotherapies in the metastatic setting. Prior anthracycline, taxane, gemcitabine, and anti-HER2 agents (i.e. trastuzumab, pertuzumab, lapatinib, neratinib, TDM-1, etc.) are allowed. If patients received prior gemcitabine, it could not have been combined with pertuzumab. Patients should have progression of disease on current therapy.
- Measurable or non-measurable disease.
- LVEF > 50%
- Hematologic parameters: white blood cell (WBC) count of > 3000/ul, absolute neutrophil count (ANC) > 1000/ul, platelets > 100,000/ul, hemoglobin > 10.0 g/dl
- Non-hematologic parameters: bilirubin < 1.5 mg/dl, AST/ALT < 2.5 x upper limit of normal (ULN), alkaline phosphatase < 5 x ULN.
- Creatinine < 1.5 mg/dl
- Patients with “treated and stable” brain lesions of a duration of > 2 months may be enrolled.

6.2 Subject Exclusion Criteria

- History of prior unstable angina, myocardial infarction, CHF, uncontrolled ventricular arrhythmias within 12 months
- History of prior > G 3 hypersensitivity (HSR) or any toxicity to trastuzumab or pertuzumab that warranted permanent cessation of this agent
- History of hepatitis B or C
- Pregnant patients

7.0 RECRUITMENT PLAN

This study is open to patients with metastatic HER2+ breast carcinoma at MSKCC or MSKCC satellites. These patients will be identified and recruited from the breast cancer patients seen at the Breast Cancer Center at MSKCC, MSKCC satellites, and Alliance affiliates.

Patients who are potentially eligible will be evaluated at the Breast Cancer Center at MSKCC or MSKCC satellites. This initial encounter will include a discussion of the proposed treatment

and the rationale for its use. Eligible patients will be required to review and sign an informed consent.

8.0 PRETREATMENT EVALUATION

- Evidence of disease evaluation within <u>4 weeks</u> prior to starting treatment:
    - 18-F FDG PET (optional).
    - Diagnostic CT chest/abdomen +/- pelvis scan OR MRI chest/abdomen +/-pelvis scan
- ECHO. When an ECHO cannot be done, a MUGA scan may be done within 4 weeks of treatment.
- EKG within 4 weeks of treatment
- History and physical examination (including vitals and ECOG performance status) within 4 weeks prior to starting treating
- Blood work within <u>2 weeks</u> prior to starting treatment
    - CBC
    - Comprehensive profile
    - Pregnancy test (serum if pre- or perimenopausal); no pregnancy test in post-menopausal women

9.0 TREATMENT/INTERVENTION PLAN

The regimen will consist of gemcitabine (1000 mg/m^2 IV weekly days 1 + 8 q 21 days) + trastuzumab (8 mg/kg loading dose followed by 6 mg/kg every 3 weeks) + pertuzumab (840 mg as a loading dose followed by 420 mg every 3 weeks), all given intravenously (IV) (+/- 3 days). Loading doses of trastuzumab and pertuzumab will not be required for patients who have received it < 6 weeks prior to Cycle 1 Day 1.Trastuzumab may be given IV weekly (4 mg/kg loading dose followed by 2 mg/kg weekly) (+/- 3 days) in lieu of the every 3 week schedule at physician's discretion.

Premedications for trastuzumab and pertuzumab should include acetaminophen 650 mg orally and diphenhydramine 25-50mg IV for the loading dose. Subsequent doses of trastuzumab and pertuzumab do not require premedications.

Patients will be on treatment until progression of disease.

Each cycle will consist of 3 weeks of HP and gemcitabine given on days 1+8.(+/- 3 days).

<u>Dose Reductions and Modifications:</u>
This study will use the NCI Common Toxicity Criteria (CTC) AE version 4.0 for toxicity.

Note: Actual weight should be used rather than ideal body weight.

Note: If a weight change of ≥ 10% occurs, the dose of trastuzumab and pertuzumab should be adjusted accordingly.

Gemcitabine:

Gemcitabine will be administered at 1000 mg/m^2 intravenously over 30 minutes on days 1 and 8 of q 21 days (+/- 3 days).

After each gemcitabine treatment, dose adjustments of gemcitabine should be based on hematologic and non-hematologic toxicities. Patients experiencing neutropenic fever (ANC <1,000/mm^3 and a single temperature ≥ 38.3°C or a sustained temperature of ≥ 38°C for more than one hour) should use filgrastim with subsequent treatments, in accordance with ASCO guidelines. If patients experience febrile neutropenia again despite the use of filgrastim, then a 20% dose reduction is allowed.

In terms of hematologic toxicities, if on the day that gemcitabine is due, and the platelet counts are <50,000/μL and/or ANC <1000/μL have not recovered to ≦ Grade 2, treatment should be delayed by ≦ 1 week and CBC and toxicity grading should be repeated. Once the platelet counts are ≥ 50,000/μL and/or ANC ≥ 1000/μL, patient can be maintained on same dose of gemcitabine and filgrastim should be used with the subsequent chemotherapy dose (in accordance with ASCO guidelines) if the delay in treatment is due to an ANC of <1000/μL. Alternatively, patients can have one gemcitabine dose reduction and filgrastim may be used at physician's discretion. If another event occurs with first dose reduction in which the platelet count is still <50,000/μL and/or ANC <1000/μL have not recovered to ≦Grade 2, a further delay of ≦ 1 week is required and CBC and toxicity grading should be repeated within ≦ 1 week. Once the platelet count is ≥ 50,000/μL and/or ANC ≥1000/μL, patient can be maintained on same dose of gemcitabine and filgrastim should be used with the subsequent chemotherapy dose (in accordance with ASCO guidelines) if the delay in treatment is due to an ANC of <1000/μL.Alternatively, gemcitabine can be re-initiated with a second dose reduction and filgrastim may be used at physician's discretion. If there is a chemotherapy postponement leading to a delay in initiating the subsequent cycle, up to 3 weeks is allowed to proceed with subsequent cycle. With initiation of the subsequent cycle, D1 of gemcitabine and HP should be synchronized or given together. For example, if D 8 gemcitabine from the previous cycle was delayed, then D1 of gemcitabine with HP of the next cycle will be started within 2 weeks after D 8 of prior cycle. Up to 3 consecutive weeks of a delay in the administration of gemcitabine is allowed. If there is a delay of > 3 consecutive weeks due to toxicities, the patient will be removed from study. A maximum of 2 dose reductions are allowed (from 1000 mg/m2 to 800 mg/m2 and from 800 mg/m2 to 600 mg/m2). If Day 1 of gemcitabine is delayed, ideally trastuzumab and pertuzumab should be delayed together in order to synchronize subsequent treatments. Alternatively, if Day 1 of gemcitabine is held (as

up to 3 weeks of treatment can be held for any reason), trastuzumab and pertuzumab may be given as scheduled without gemcitabine.

In terms of non-hematologic toxicities, if on the day that gemcitabine is due and ≧ Grade 3 non-hematologic toxicities have not recovered to ≦ Grade 2, treatment may be delayed up to 3 weeks. Once the patient is re-started on gemcitabine, a dose reduction is allowed. If ≧ Grade 3 non-hematologic toxicities occur again with one dose reduction, another dose reduction is allowed. If there is a delay of > 3 consecutive weeks due to toxicities, the patient will be removed from study. A maximum of 2 dose reductions are allowed (from 1000 mg/m2 to 800 mg/m2 and from 800 mg/m2 to 600 mg/m2). If Day 1 of gemcitabine is delayed, ideally trastuzumab and pertuzumab should be delayed together in order to synchronize subsequent treatments.

Note: For those who need GCSF support, pegfilgrastim may be used but only if there is a 2 week interval between each chemotherapy dose. For example, pegfilgrastim may be give between day 8 and day 1 of next cycle as each cycle is every 21 day.

Note: At 3 months if the patient is deemed to have responded to the combination of gemcitabine, trastuzumab, and pertuzumab, at the physician's discretion, the chemotherapy may be stopped and the patient may continue on trastuzumab and pertuzumab alone. Alternatively, chemotherapy frequency and the dosing may be altered at physician's discretion. At a later point, if the patient has progression of disease on HP alone, the same chemotherapy may be added back. After baseline and week 12 ECHO (+/- 7 days), ECHO (or MUGA) may be ordered infrequently at physician's discretion. This is based on a large body of evidence and from our study (IRB #10-142) that pertuzumab with trastuzumab is associated with only rare rates of grade 3-4 LVSD (symptomatic heart failure) in the order of 0-1%. If patients had gemcitabine re-initiated due to progression of disease, the interval between CT or MRI of chest/abdomen +/- pelvis and/or optional FDG PET scans is at the physician's discretion. It does not have to be every 12 weeks as the patient would have already met the 3-month PFS endpoint.

Trastuzumab:

Trastuzumab loading dose (8 mg/kg) IV will be administered on same day as day 1 of cycle 1 of chemotherapy and is continued every 3 weeks at 6 mg/kg (+/- 3 days). Trastuzumab may be given weekly (4 mg/kg loading dose →2 mg/kg weekly). There is no dose modification for trastuzumab. A loading dose of trastuzumab will not be required for patients who have received it < 6 weeks prior to Cycle 1 Day 1.

Pertuzumab:

Pertuzumab loading dose (840 mg) IV will be administered on same day as day 1 of cycle 1 of chemotherapy and is continued every 3 weeks at 420 mg (+/- 3 days). There is no dose modification for pertuzumab. A loading dose of pertuzumab will not be required for patients who have received it < 6 weeks prior to Cycle 1 Day 1.

Note: Vital signs should be monitored per institutional guidelines.

Note: Patients will have both trastuzumab and pertuzumab held for significant asymptomatic LVEF declines as outlined in the table below.

RELATIONSHIP OF LVEF TO THE LOWER LIMIT OF NORMAL (LLN)	ABSOLUTE DECREASE OF < 10 PERCENTAGE POINTS	ABSOLUTE DECREASE OF 10 TO 15 PERCENTAGE POINTS	ABSOLUTE DECREASE OF ≥ 16 PERCENTAGE POINTS
**Within normal limits**	Continue H + P	Continue H + P	Hold H + P and repeat LVEF within 3 weeks
**1 to 5 percentage points below the LLN**	Continue H + P	Hold H + P and repeat LVEF within 3 weeks	Hold H + P and repeat LVEF within 3 weeks
**≥ 6 percentage points below the LLN**	Continue H + P and repeat LVEF within 3 weeks	Hold H+ P and repeat LVEF within 3 weeks	Hold H + P and repeat LVEF within 3 weeks

H=Trastuzumab

P=Pertuzumab

A repeated LVEF may be assessed by an ECHO or MUGA

Rules for interpreting and applying “repeat” LVEF results:

- H+ P must be permanently discontinued when two consecutive “hold” categories occur.
- H+P must be permanently discontinued when three intermittent “hold” categories occur. (At the investigator’s discretion, H + P may also be permanently discontinued prior to the occurrence of three intermittent “hold” categories.)
- If LVEF is maintained at a “continue and repeat LVEF” or improves from a “hold” to a “continue and repeat LVEF” category, an additional ECHO (or MUGA scan) prior to the next scheduled LVEF assessment will be at the investigator’s discretion.
- Patient who experiences a significant “asymptomatic” LVEF decline while on trastuzumab + pertuzumab which result in permanent discontinuation of H + P will be seen in the cardiology clinic.
- If a patient experiences G 3-4 symptomatic congestive heart failure at any time, trastuzumab and pertuzumab should be stopped and she/he will be removed from study and be seen by a cardiologist.

Note: If a patient has any temporary or permanent cessation of trastuzumab and pertuzumab due to asymptomatic or symptomatic LVEF declines, weekly chemotherapy may be continued as planned.

Diarrhea and Rash Management

Pertuzumab may cause diarrhea and rash. Below are guidelines in place for management of these toxicities as follows:

Pertuzumab Diarrhea and Rash Management

**Diarrhea**		
**Grade**	**Management**	**Dose Delay**
1	Loperamide<br>at 1st onset	None
2	Loperamide<br>at 1st onset	None;<br>If unacceptable, hold pertuzumab ≤ 21 days, resume pertuzumab at same dose *
≥ 3	Loperamide<br>at 1st onset	Hold pertuzumab ≤ 21 days until ≤ G 1, resume pertuzumab at same dose **

**Rash**		
**Grade**	**Management**	**Dose Delay**
1	minocycline, topical tetracycline or clindamycin,	None
	topical silver sulfadiazine, diphenhydramine, oral prednisone	

2	minocycline, topical tetracycline or clindamycin, topical silver sulfadiazine, diphenhydramine, oral prednisone	None;<br><br>If unacceptable, hold pertuzumab ≤ 21 days, resume same dose #
≥ 3	Same as above	Hold pertuzumab ≤ 21 days until ≤ G 1, resume at same dose ##

<u>Note:</u> Refer to **Appendix B** for diarrhea and rash management.

* If the patient experiences a G 2 diarrhea that is "unacceptable", hold pertuzumab for ≤ 21 days and resume at the same dose. If G 2 diarrhea recurs that is "unacceptable" despite a held dose previously for G 2 toxicity, then can hold pertuzumab again for ≤ 21 days. Trastuzumab should be continued as planned. There is no dose reduction for trastuzumab and pertuzumab.

** If the patient experiences G ≥ 3 diarrhea, hold pertuzumab for ≤ 21 days until ≤ G 1 and then resume pertuzumab at the same dose. If > 3 weeks are required for the toxicity to reach to ≤ G 1, then pertuzumab should be held permanently and the patient should be taken off the study (The patient may remain on chemotherapy and trastuzumab at the physician's discretion). If G 3 diarrhea recurs upon being re-challenged with pertuzumab, then the patient will be removed from study. There is no dose reduction for trastuzumab and pertuzumab.

If the patient experiences a G 2 rash that is "unacceptable", hold pertuzumab for ≤ 21 days and resume at the same dose. If G 2 rash recurs that is "unacceptable" despite a held dose previously for G 2 toxicity, then can hold pertuzumab again for < 21 days. Trastuzumab and paclitaxel should be continued as planned. There is no dose reduction for trastuzumab and pertuzumab.

If the patient experiences G ≥ 3 rash, hold pertuzumab ≤ 21 days until ≤ G 1 and then resume pertuzumab at the same dose. If > 3 weeks are required for the toxicity to reach to ≤ G 1, then pertuzumab should be held permanently and the patient should be taken off the study (The patient may remain on chemotherapy and trastuzumab at the physician's discretion). If G 3 rash recurs upon being re-challenged with pertuzumab, then the patient will be removed from study. There is no dose reduction for trastuzumab and pertuzumab.

Note: If the patient is found to have brain metastasis that requires treatment but is responding to this study regimen systemically, she may be allowed to take a break from receiving chemotherapy for up to 6 weeks in order to have treatment for brain metastasis (i.e., whole brain radiation, stereotactic radiation, surgical resection, etc). It is encouraged that trastuzumab and pertuzumab should be continued every 3 weeks if possible. As it is not known that chemotherapy, trastuzumab, or pertuzumab can cross the blood

brain barrier well, a central nervous system progression will not be considered a treatment failure. This will be the one time that a patient may have up to a 6 week break from chemotherapy while undergoing treatment for brain metastasis.

10.0 EVALUATION DURING TREATMENT/INTERVENTION

- Patients will be seen at every cycle of treatment preferably on day 1 of each cycle. If there is a schedule conflict, patients may be seen on day 8. There is a +/- 3 day window. Each evaluation will consist of the following:
    - History and physical examination with vital signs, ECOG performance status.
    - Recording of the adverse events
    - Labs
        - CBC prior to each chemotherapy infusion (A CBC can be completed up to 3 days prior to the infusion).
        - Comprehensive profile once per cycle of treatment. The patient does not have to wait for the result to come back to start treatment on the same day.
        - If the patient is deemed to have met the 3-month PFS endpoint and is on a chemotherapy holiday and is on HP alone, then CBC and comprehensive labs will be done only at physician's discretion. During this period, the patient is only required to be seen every other cycle.
- Patients may have their treatment schedule modified as follows:
    - Patients may have a delay of treatment or treatment held for up to 3 weeks (for any reason).
- Patients will be assessed radiographically at 12 weeks (+/- 7 days) from cycle 1 day 1 and then every 12 weeks (+/- 7 days) afterwards. Radiographic assessment will include:
    - 18F FDG PET (optional)
    - Diagnostic CT chest/abdomen +/- pelvis scan OR MRI chest/abdomen +/-pelvis scan

- Patients will have an ECHO at 12 weeks (+/- 7 days) from cycle 1 day 1. If an ECHO cannot be done, a MUGA scan may be done. The same modality should be used throughout the study. After 12 weeks, ECHO can be ordered at the treating physician's discretion.

	**Pre- Treatment**	**Prior to each chemo- therapy treatment [3]**	**Every Cycle +/- 3 days**	**Every 12 weeks**	**After Treatment Completion [4]**

**Medical History**	X (w/i 4 weeks)		X		X
**Physical examination**	X (w/i 4 weeks)		X		X
**ECOG PS**	X (w/i 4 weeks)		X		
**CBC**	X (w/i 2 weeks)	X	X		
**Comprehensive Profile**	X (w/i 2 weeks)		X		
**EKG**	X (w/i 4 weeks)				
**LVEF** [1]	X (w/i 4 weeks)				
**Pregnancy test**	X (w/i 2 weeks)				
**Evidence of disease evaluation (EOD)** [2]	X (w/i 4 weeks)			X	
**Signed informed consent**	X (w/i 4 weeks)				
**Adverse event/toxicity assessment**			X		

1. LVEF is assessed via an ECHO. If an ECHO cannot be done, a MUGA scan may be done. The LVEF will be assessed at 12 weeks (+/- 7 days) from cycle 1 day 1. Subsequent LVEF assessment is at the discretion of the treating physician.
2. EOD evaluation will consist of a 18F FDG PET/CT (optional) with diagnostic CT or MRI. EOD evaluation will be assessed at 12 weeks (+/- 7days) from cycle 1 day 1and every 12 weeks (+/- 7 days) afterwards.
3. Prior to the first cycle of treatment, weight must be obtained.
4. After treatment completion, the patient will be seen for routine follow-ups (i.e., medical history, physical examination) at the discretion of the treating physician.

11.0 TOXICITIES/SIDE EFFECTS

Embryo-fetal toxicity. There are no clinical studies of pertuzumab or trastuzumab in pregnant women. IgGs are known to cross the placental barrier. Nonclinical reprotoxicity data in cynomolgus monkeys treated with pertuzumab showed embryofetal losses, oligohydramnios, and renal hypoplasia (please refer to the pertuzumab investigator’s brochure for details). In the postmarketing setting, oligohydramnios has been reported in women who received trastuzumab during pregnancy,

either in combination with chemotherapy or as a single agent. Neither pertuzumab nor trastuzumab should be used during pregnancy.

It is not known whether trastuzumab or pertuzumab is excreted in human milk. Because maternal IgG is excreted in milk and either monoclonal antibody could harm infant growth and development, women should be advised to discontinue nursing during pertuzumab or trastuzumab therapy and not to breastfeed for at least seven months following the last dose of either monoclonal antibody.

For women of childbearing potential (who have not undergone surgical sterilization), and the female partners of male participants; agreement must be obtained to use highly effective contraception.

On the basis of pharmacokinetic considerations, contraception methods should start a minimum of 14 days prior to the first administration of study drug and continue for the duration of study treatment and for at least 7 months after the last dose of study treatment (please refer to trastuzumab investigator's brochure for details).

Encourage pregnant women with breast cancer who are exposed to trastuzumab or pertuzumab to enroll in the MotHER Pregnancy Registry (phone 1-800-690-6720).

Trastuzumab (Herceptin®)

Infusion-Associated Symptoms. During the first infusion with Herceptin, a symptom complex consisting of chills and/or fever is observed in approximately 40% of patients. Other signs and/or symptoms may include nausea, vomiting, pain, rigors, headache, cough, dizziness, rash, and asthenia. These symptoms are usually mild to moderate in severity, and occur infrequently with subsequent Herceptin infusions. These symptoms can be treated with an analgesic/antipyretic such as meperidine or paracetamol, or an antihistamine such as diphenhydramine.

Serious Infusion-Associated Events. Serious adverse reactions to Herceptin infusion including dyspnea, hypotension, wheezing, bronchospasm, tachycardia, reduced oxygen saturation and respiratory distress have been reported infrequently. In rare cases (4 per 10,000), these events were associated with a clinical course culminating in a fatal outcome. Serious reactions have been treated with supportive therapy such as oxygen, beta-agonists, corticosteroids and withdrawal of Herceptin as indicated.

Hematologic Toxicity. In the clinical trials, an increased incidence of anemia was observed in patients receiving Herceptin plus chemotherapy compared with patients receiving chemotherapy alone. The majority of these anemia events were mild or moderate in intensity and reversible; none resulted in discontinuation of Herceptin therapy. In the clinical trials, the per-patient incidences of moderate to severe neutropenia and of febrile neutropenia were higher in patients receiving Herceptin in combination with myelosuppressive chemotherapy as compared to those who received chemotherapy alone. In the post marketing setting, deaths due to sepsis in patients with severe neutropenia have been reported in patients receiving Herceptin and myelosuppressive chemotherapy, although in controlled clinical trials (pre- and post-marketing), the incidence of septic deaths

was not significantly increased. The pathophysiologic basis for exacerbation of neutropenia has not been determined; the effect of Herceptin on the pharmacokinetics of chemotherapeutic agents has not been fully evaluated. The observed incidence of leukemia among Herceptin-treated patients appears to be consistent with the expected incidence of leukemia among patients treated with chemotherapy for metastatic breast cancer. Therefore, the contribution of Herceptin to the etiology of acute leukemia or myelodysplastic syndrome in these cases is unclear.

Pertuzumab

Diarrhea and rash

Although pertuzumab targets HER2, because of its role in heterodimerization with other members of the HER family (i.e., HER1/EGFR), it may cause toxicities associated with the use of EGFR inhibitors. Diarrhea and rash are common events increased with pertuzumab given in combination with chemotherapy compared with chemotherapy alone. Diarrhea has been reported in 60-70% of patients treated with pertuzumab and was mostly of grades 1-2. The mechanism of diarrhea and rash are unknown, but the nature is similar to that of other agents causing HER1 inhibition. In the event of diarrhea, early intervention with anti-diarrhea medication should be considered and patients treated with fluid and electrolyte replacement, as clinically indicated.

Infusion-related Symptoms

Monoclonal antibodies may cause infusion-associated symptoms such as fever, chills, hypotension, shortness of breath, skin rash, headache, nausea, and/or vomiting. Serious or severe infusion-related symptoms have been rarely observed with 7 patients experiencing serious reactions to date [hypersensitivity, fatal ARDS, pulmonary edema, anaphylaxis, dyspnea with hypotension, infusion-related reaction (unsteady on feet, headache)]. In pertuzumab single-agent studies, less than 5% of patients experienced adverse reactions during pertuzumab infusions. Serious infusion-related reactions with pertuzumab have been infrequently reported (< 1%). Intravenous pertuzumab administration should be performed in the setting with emergency equipment and staff who re trained to monitor medical situations and respond to medical emergencies. Patients should be monitored during each pertuzumab infusion for any adverse effects, as infusion reactions may occur with the first or subsequent doses. The infusion should be stopped for patients who develop dyspnea, clinically significant hypotension, or other clinically significant events. Patients who experience an NCI-CTC grade 4 allergic reaction or acute respiratory distress syndrome should not receive additional pertuzumab.

Cardiac Toxicities

Since pertuzumab targets HER2, as with trastuzumab, there is a potential risk of cardiac dysfunction, particularly in patients who have received prior anthracycline treatment. Cardiac toxicities, predominantly asymptomatic LVEF declines, and 4 cases of cardiac failure have been reported in approximately 840 patients with advanced malignant disease or early stage

HER2 (+) breast cancer treated with pertuzumab. Two of these cases occurred in patients with metastatic breast cancer who had received prior anthracyclines and two in patients with ovarian cancer. No clear association between the frequency, nature, and severity of pertuzumab-related toxicities and dose level has been observed.

Patients with significant cardiac disease or baseline LVEF below the institution's lower limit of normal (LLN) should not commence treatment with pertuzumab. Risk factors for pertuzumab-associated cardiac dysfunction are not known at this time. This risk should be carefully weighed against the potential benefit in patients who have received prior anthracyclines. Monitoring of the LVEF is advised while patients are receiving pertuzumab. If symptomatic LVEF decline develops (NCI-CTC grade 3 or 4), the patient must discontinue pertuzumab. Left ventricular dysfunction, symptomatic or not, should be treated and followed according to standard medical practice.

Other Side-effects

Other side-effects of pertuzumab include low blood count and fatigue.

<u>Gemcitabine</u>

Hematologic — In studies <1% of patients discontinued therapy for either anemia, leukopenia, or thrombocytopenia. Red blood cell transfusions were required by 19% of patients. The incidence of sepsis was less than 1%. Petechiae or mild blood loss (hemorrhage), from any cause, was reported in 16% of patients; less than 1% of patients required platelet transfusions. Patients should be monitored for myelosuppression during Gemzar therapy and dosage modified or suspended according to the degree of hematologic toxicity.

Gastrointestinal — Nausea and vomiting were commonly reported (69%) but were usually of mild to moderate severity. Severe nausea and vomiting (WHO Grade 3/4) occurred in <15% of patients. Diarrhea was reported by 19% of patients, and stomatitis by 11% of patients.

Hepatic — In clinical trials, Gemzar was associated with transient elevations of one or both serum transaminases in approximately 70% of patients, but there was no evidence of increasing hepatic toxicity with either longer duration of exposure to Gemzar or with greater total cumulative dose. Serious hepatotoxicity, including liver failure and death, has been reported very rarely in patients receiving Gemzar alone or in combination with other potentially hepatotoxic drugs .

Renal — In clinical trials, mild proteinuria and hematuria were commonly reported. Clinical findings consistent with the Hemolytic Uremic Syndrome (HUS) were reported in 6 of 2429 patients (0.25%) receiving Gemzar in clinical trials. Four patients developed HUS on Gemzar therapy, 2 immediately posttherapy. The diagnosis of HUS should be considered if the patient develops anemia with evidence of microangiopathic hemolysis, elevation of bilirubin or LDH, reticulocytosis, severe thrombocytopenia, and/or evidence of renal failure (elevation of serum creatinine or BUN). Gemzar therapy should be discontinued immediately. Renal failure may not be reversible even with discontinuation of therapy and dialysis may be required.

Fever — The overall incidence of fever was 41%. This is in contrast to the incidence of infection (16%) and indicates that Gemzar may cause fever in the absence of clinical infection. Fever was frequently associated with other flu-like symptoms and was usually mild and clinically manageable.

Rash — Rash was reported in 30% of patients. The rash was typically a macular or finely granular maculopapular pruritic eruption of mild to moderate severity involving the trunk and extremities. Pruritus was reported for 13% of patients.

Pulmonary — In clinical trials, dyspnea, unrelated to underlying disease, has been reported in association with Gemzar therapy. Dyspnea was occasionally accompanied by bronchospasm. Pulmonary toxicity has been reported with the use of Gemzar. The etiology of these effects is unknown. If such effects develop, Gemzar should be discontinued. Early use of supportive care measures may help ameliorate these conditions.

Edema — Edema (13%), peripheral edema (20%), and generalized edema (<1%) were reported. Less than 1% of patients discontinued due to edema.

Flu-like Symptoms — Flu syndrome< was reported for 19% of patients. Individual symptoms of fever, asthenia, anorexia, headache, cough, chills, and myalgia were commonly reported. Fever and asthenia were also reported frequently as isolated symptoms. Insomnia, rhinitis, sweating, and malaise were reported infrequently. Less than 1% of patients discontinued due to flu-like symptoms.

Infection — Infections were reported for 16% of patients. Sepsis was rarely reported (<1%).

Alopecia — Hair loss, usually minimal, was reported by 15% of patients.
Neurotoxicity — There was a 10% incidence of mild paresthesias and a <1% rate of severe paresthesias.

Extravasation — Injection-site related events were reported for 4% of patients. There were no reports of injection site necrosis. Gemzar is not a vesicant.

Allergic — Bronchospasm was reported for less than 2% of patients. Anaphylactoid reaction has been reported rarely. Gemzar should not be administered to patients with a known hypersensitivity to this drug.

Cardiovascular — During clinical trials, 2% of patients discontinued therapy with Gemzar due to cardiovascular events such as myocardial infarction, cerebrovascular accident, arrhythmia, and hypertension. Many of these patients had a prior history of cardiovascular disease.

12.0 CRITERIA FOR THERAPEUTIC RESPONSE/OUTCOME ASSESSMENT

The primary aim of this study will be to assess the 3-month PFS under treatment with gemcitabine + HP in patients with HER2 (+) MBC with prior HP- or P-based treatment but no more than 3 prior chemotherapy regimens for MBC. Patients with measurable or non-

measurable lesions are included in this study. Response to treatment will be determined using both RECIST and PRC criteria. PRC criteria will be exploratory. Measurable lesions are defined as those that can be measured accurately in at least one diameter, that is 20 mm using conventional imaging techniques (including incremental CT) or 10 mm using spiral CT equipment. Non- measurable lesions include bony metastases, leptomeningeal disease, ascites, pleural/pericardial effusions, inflammatory breast cancer, lymphangitis carcinomatosis, and heavily calcified and cystic/necrotic lesions.

All clinical studies will take place at MSKCC including satellite locations. Patients will have an evidence of disease evaluation (EOD) evaluation at 12 weeks (+/- 7 days) from cycle 1 day 1and every 12 weeks (+/- 7 days) afterwards, which will consist of CT or MRI of chest and abdomen +/- pelvis. The 18-F FDG PET/CT scan will be optional. Evaluable patients include all patients who started therapy and for whom we are able to assess PFS status at 3 months. Patients who withdraw before 3 months for non-toxicity reasons (i.e., personal reasons, non-cancer related illness, and non-compliance) are considered inevaluable. We do not anticipate a significant early withdrawal for non-toxicity reasons. Patients who discontinue therapy due to toxicity before the 3 month endpoint, who initiate a subsequent nonprotocol therapy prior to progression, will be considered evaluable and their progression date listed as the day the subsequent therapy is initiated.
The primary endpoint is PFS and secondary endpoint will include the response rate using the RECIST criteria (version 1.1). The definitions are included as below:

- Progression-free survival (PFS) is defined from time from treatment assignment to disease progression or death, whichever comes first.
- Partial response (PR) is at least a 30% reduction in the sum of diameters of target lesions, taking as reference the baseline sum diameters.
- Complete response (CR) is the disappearance of all target lesions. Any pathological lymph nodes (whether target or non-target) must have reduction in short axis to < 10 mm.
- Stable disease (SD) is neither sufficient shrinkage to qualify for PR nor sufficient increase to qualify for PD, taking as reference the smallest sum diameters while on study.
- Progressive disease (PD) requires a 20% increase in the sum of diameters of target lesions, taking as reference the smallest sum on study (this includes the baseline sum if that is the smallest on study). In addition to the relative increase of 20%, the sum must also demonstrate an absolute increase of at least 5 mm. (Note: the appearance of one or more new lesions is also considered progression).

PET Response Criteria
Up to 5 lesions will be followed and the aggregate % change in SUVmax will be measured and the results of this will be compared to RECIST results.

13.0 CRITERIA FOR REMOVAL FROM STUDY

1) > 3 consecutive weeks of a delay in treatment due to toxicities
2) Progressive disease
3) Unacceptable toxicity
4) Intercurrent, non-cancer related illness that prevents continuation of protocol therapy or follow-up
5) Major protocol violation that would render the patient inevaluable for efficacy
6) Repeated non-compliance by the patient with protocol requirements
7) Changes in the patient's condition or study drug related toxicity such that, in the opinion of the investigator, continued participation in the protocol would compromise patient well-being
8) Withdrawal of patient's consent for personal reasons
9) Death

Patients removed due to criteria listed in 5, 6, and 7 are considered inevaluable for the primary 3 month PFS endpoint.

14.0 BIOSTATISTICS

This is a single arm phase II trial of gemcitabine/HP in patients with metastatic HER2-positive breast cancer. Patients with HER2 (+) breast cancer will be eligible if they have had prior treatment with HP- or P-based therapy and ≤ 3 prior chemotherapies in the metastatic setting. The primary objective of this trial is to determine the efficacy of this regimen. The primary endpoint of this study will be progression-free survival at 3months. Patients who are considered progression-free at 3 months are deemed treatment successes. Treatment failures are those patients whose disease progressed or who died before the 3 month mark, or who discontinued study participation due to adverse events related to therapy. Patients who discontinue study participation prior to 3 months for reasons clearly not due to study therapy or disease progression may be considered ineligible for response and not included in efficacy analysis. Patients who discontinue therapy due to toxicity before the 3 month endpoint, who initiate a subsequent nonprotocol therapy prior to progression, will be considered evaluable and their progression date listed as the day the subsequent therapy is initiated.

A Simon Optimal 2-stage design will be used to evaluate the efficacy of this regimen. A large body of evidence in 3 studies involving over 900 patients, who were heavily pretreated, demonstrated that standard options led to a median PFS of 3-4 months. [32, 40-41] In our study we are seeking to determine if HP + gemcitabine will be render a better median PFS than 3 months. We hypothesize that about 70% of patients will be progression-free at 3 months. To see a difference in the proportion of patients who are alive and progression free at 3 months from 50% to 70%, we will enroll 21 patients in the first stage. If 12 or more are alive and progression free at 3 months, the trial will accrue to the second stage for a total of 45 patients, although accrual will not be halted temporarily while the first stage patients are evaluated. If 27 or more patients out of the 45 patients are alive and progression free, the trial will be deemed a success. This design assumes a 10% type I and type II error.

We will consider a symptomatic congestive heart failure rate (CHF) rate of ≤ 4% as acceptable to be consistent with what has been reported in previous trials with trastuzumab alone (symptomatic CHF rates ranging from 2-4%). For this study, to be homologous to the large phase III trial, CLEOPATRA, a cardiac event is defined as a) "symptomatic" left ventricular systolic dysfunction (LVSD) (deaths and non-deaths), b) non-LVSD cardiac death, or c) probable cardiac death. All patients who receive any amount of study therapy (i.e. pertuzumab) will be included in the analyses of toxicity and tolerability. We do not anticipate a high incidence of CHF based on our experience with a previous study of taxane/HP (IRB # 10-142), as there was no incidence of CHF in our patient population. Of note from the CLEOPATRA study and from our study (IRB #10-142), pertuzumab with trastuzumab is associated with only rare rates of grade 3-4 LVSD (symptomatic heart failure) in the order of 0-1%.

Upon completion of the study, the 3 month PFS rate will be estimated and a 90% exact confidence interval will be constructed. Progression-free survival and median overall survival will also be estimated by the Kaplan-Meier method. Selected non-hematologic and hematologic toxicities will be described by frequency and grade, by each cycle and all cycles, with the maximum grade over all cycles used as the summary measure per patient (CTCAE v4). In terms of the exploratory endpoints, response by PRC on 18-F FDG-PET will be compared with response by RECIST on dedicated CT and descriptive data will be reported for patients who completed the optional FDG-PET scans.

Based on our experience, we anticipate an accrual rate of 2-3 patients per month over the course of 2 years. Additional patients will be accrued to take place of in-evaluable patients as necessary. We expect this early dropout to be a rare event, occurring 5% of the time, and will be examined closely as a source for potential bias.

15.0 RESEARCH PARTICIPANT REGISTRATION AND RANDOMIZATION PROCEDURES

15.1 Research Participant Registration

Confirm eligibility as defined in the section entitled Criteria for Patient/Subject Eligibility.

Obtain informed consent, by following procedures defined in section entitled Informed Consent Procedures.

During the registration process registering individuals will be required to complete a protocol specific Eligibility Checklist.

All participants must be registered through the Protocol Participant Registration (PPR) Office at Memorial Sloan-Kettering Cancer Center. PPR is available Monday through Friday from 8:30am – 5:30pm at 646-735-8000. Registrations must be submitted via the PPR Electronic

Registration System (http://ppr/). The completed signature page of the written consent/verbal script and a completed Eligibility Checklist must be faxed to PPR.

15.2 Randomization

Randomization will not take place in this trial.

16.0 DATA MANAGEMENT ISSUES

A Research Study Assistant (RSA) will be assigned to the study. The responsibilities of the RSA include project compliance, data collection, abstraction and entry, data reporting, regulatory monitoring, problem resolution and prioritization, and coordinate the activities of the protocol study team.

The Clinical Research Database (CRDB) will be used for data collection. The data will be reported to the institution (IRB) and the sponsor (Roche/Genentech) as appropriate. Documentation linking patient identifiers and patient samples and results will be securely maintained in the CRDB with access limited to study investigators.

16.1 Quality Assurance

Weekly registration reports will be generated to monitor patient accruals, protocol compliance, eligibility verification, informed consent procedure, data accuracy, and completeness of registration data. Routine data quality reports will be generated to assess missing data and inconsistencies. Accrual rates and extent and accuracy of evaluations and follow-up will be monitored periodically throughout the study period and potential problems will be brought to the attention of the study team for discussion and action.

Random-sample data quality and protocol compliance audits will be conducted by the study team, at a minimum of two times per year, more frequently if indicated.

16.2 Data and Safety Monitoring

The CRDB will be the only method of capturing the data. The Data and Safety Monitoring (DSM) Plans at Memorial Sloan-Kettering Cancer Center were approved by the National Cancer Institute in September 2001. The plans address the new policies set forth by the NCI in the document entitled "Policy of the National Cancer Institute for Data and Safety Monitoring of Clinical Trials" which can be found at: http://cancertrials.nci.nih.gov/researchers/dsm/index.html. The DSM Plans at MSKCC were established and are monitored by the Office of Clinical Research. The MSKCC Data and Safety Monitoring Plans can be found on the MSKCC Intranet at: https://one.mskcc.org/sites/pub/clinresearch/Documents/MSKCC%20Data%20and%20Safety%20Monitoring%20Plans.pdf

There are several different mechanisms by which clinical trials are monitored for data, safety and quality. There are institutional processes in place for quality assurance (e.g., protocol monitoring, compliance and data verification audits, therapeutic response, and staff education on clinical research QA) and departmental procedures for quality control, plus there are two institutional committees that are responsible for monitoring the activities of our clinical trials programs. The

committees: *Data and Safety Monitoring Committee (DSMC)* for Phase I and II clinical trials, and the *Data and Safety Monitoring Board (DSMB)* for Phase III clinical trials, report to the Center's Research Council and Institutional Review Board.

17.0 PROTECTION OF HUMAN SUBJECTS

Prior to the enrollment of each patient, the risks, benefits and objectives of the study will be reviewed with the participant, including a discussion of the possible toxicities and side effects. Every effort will be made to keep study records private. Neither the patient's name nor anything else that could identify the patient will be used in any reports or publications that result from this study. Trained staff at Memorial Hospital, the Food and Drug Administration, or the study supporters will be able to review the medical records if necessary. The patient may terminate her participation in the study at any time during the trial.

Prior to the enrollment of each patient, the risks, benefits and objectives of the study will be reviewed with the participant, including a discussion of the possible toxicities and side effects. Alternative, non-protocol, treatment options will be discussed with the patient. It will be reviewed that participation in this clinical trial is voluntary and that the patient may withdraw consent at any time. The study is designed with careful safety monitoring for toxicity including physician visits. Specific guidelines for symptom management are in place to protect the study participant.

Consent process: All patients at MSKCC who meet the inclusion criteria will be eligible. Participation in the trial is voluntary. All patients will be required to sign a statement of informed consent, which must conform to IRB guidelines. The informed consent procedure is described in **Section 18.0**.

Potential Risks: Our eligibility criteria and screening procedures are established to exclude individuals for whom this study treatment is not appropriate. Our screening procedures begin with medical chart review to identify any individuals with any condition or reasons that may prohibit study entry followed by oncologist approval to screen/identify patients who may not be eligible for any additional reasons. Finally, in-person assessments will be performed to screen/identify patients.

Risks of research participation: The greatest risk is release of information from health or research records in a way that violates privacy rights. MSKCC will protect records so that name, address, phone number, and any other information that identifies the participant will be kept private. It will be stated to the participant that the chance that this information will be given to an unauthorized individual without the participant's permission is very small.

Costs/compensation: Patients will be charged for physician visits, routine laboratory tests and radiologic studies required for monitoring their condition. The patients will not be billed for any study-related procedures. The participant is informed that there are no plans to provide

financial compensation for use of their human biologic specimens, nor are there plans for the participant to receive money for any new products, tests, and discoveries that might come from this research.

Alternatives: The alternative to this trial would be not to participate in the study and receive routine standard of care.

Confidentiality: Every effort will be made to maintain patient confidentiality. Research and hospital records are confidential. Patients' names and any other identifying information will not be used in reports or publications resulting from this study. Other authorized agencies and appropriate internal personnel (eg. qualified monitors from MSKCC) and external personnel, its authorized agents, the FDA, and/or other governmental agencies) may review patient records as required.

Patient safety: Patients are monitored by physicians and oncology nurses who are very familiar with clinical trials. In the case of an adverse reaction, immediate medical attention is available. In the evenings and weekends, we have a 24-hour urgent care facility for outpatients. The PI will also be available at all times to organize any necessary intervention.

Monitoring of data to ensure safety: This study is to be monitored by the institutional IRB. This incorporates an independent data and safety monitoring board established by arrangement with the National Cancer Institute. The analysis of safety will include all patients. Adverse events, including all toxic effects of treatment, will be tabulated individually, and summarized by severity and causality.

Voluntariness of research participation: It is stated that taking part in this study is voluntary and patients have the right to withdraw at any time. Participation in the study will not impact on the clinical care patients receive.

Withdrawal: A note-to-file documenting the patient's withdraw must be filed in his/her EMR.

17.1 Privacy

MSKCC's Privacy Office may allow the use and disclosure of protected health information pursuant to a completed and signed Research Authorization form. The use and disclosure of protected health information will be limited to the individuals described in the Research Authorization form. A Research Authorization form must be completed by the Principal Investigator and approved by the IRB and Privacy Board (IRB/PB).

17.2 Serious Adverse Event (SAE) Reporting

An adverse event is considered serious if it results in ANY of the following outcomes:

- Death

- A life-threatening adverse event
- An adverse event that results in inpatient hospitalization or prolongation of existing hospitalization
- A persistent or significant incapacity or substantial disruption of the ability to conduct normal life functions
- A congenital anomaly/birth defect
- Important Medical Events (IME) that may not result in death, be life threatening, or require hospitalization may be considered serious when, based upon medical judgment, they may jeopardize the patient or subject and may require medical or surgical intervention to prevent one of the outcomes listed in this definition

Note: Hospital admission for a planned procedure/disease treatment is not considered an SAE.

SAE reporting is required as soon as the participant signs consent. SAE reporting is required for 30-days after the participant's last investigational treatment or intervention. Any events that occur after the 30-day period and that are at least possibly related to protocol treatment must be reported.

If an SAE requires submission to the IRB office per IRB SOP RR-408 'Reporting of Serious Adverse Events', the SAE report must be sent to the IRB within 5 calendar days of the event. The IRB requires a Clinical Research Database (CRDB) SAE report be submitted electronically to the SAE Office as follows:

Reports that include a Grade 5 SAE should be sent to saegrade5@mskcc.org. All other reports should be sent to sae@mskcc.org.

The report should contain the following information:

Fields populated from CRDB:

- Subject's initials
- Medical record number
- Disease/histology (if applicable)
- Protocol number and title

Data needing to be entered:

- The date the adverse event occurred
- The adverse event
- The grade of the event
- Relationship of the adverse event to the treatment (drug, device, or intervention)
- If the AE was expected
- The severity of the AE
- The intervention

- Detailed text that includes the following
    - A explanation of how the AE was handled
    - A description of the subject's condition
    - Indication if the subject remains on the study
- If an amendment will need to be made to the protocol and/or consent form
- If the SAE is an Unanticipated Problem

The PI's signature and the date it was signed are required on the completed report.

17.2.1 Reporting SAEs:

All SAEs that are serious and reasonably or probably related to the use of pertuzumab (this applies to both expected and unexpected events) should be recorded on an MSK CRDB SAE report. The completed Medwatch/case report should be faxed immediately upon completion and faxed as soon as possible to:

Roche/Genentech Drug Safety

Pertuzumab Safety Monitor

Tele: 1-888-835-2555

Fax: (650) 225-4682 or (650) 225-4683

AND:

Study Coordination Center/Principal Investigator: Chau Dang, M.D.

Contact Information phone: 646-888-4554 fax : 646-888-4555

- Relevant follow-up information should be submitted to Genentech Drug Safety as soon as it becomes available.
- Serious AE reports that are related to the [Pertuzumab, Trastuzumab] and AEs of Special Interest (regardless of causality) will be transmitted to Genentech within fifteen (15) calendar days of the Awareness Date.

- Pertuzumab, Trastuzumab Additional Reporting Requirements to Genentech include the following:
- Any reports of pregnancy following the start of administration with the [Pertuzumab, Trastuzumab] will be transmitted to Genentech within thirty (30) calendar days of the Awareness Date.
- All Non-serious Adverse Events originating from the Study will be forwarded in a quarterly report to Genentech.

Note: Investigators should also report events to their IRB as required.

Occasionally Roche/Genentech may contact the reporter for additional information, clarification, or current status of the subject for whom and adverse event was reported. For questions regarding SAE reporting, you may contact the Roche/Genentech Drug Safety

representative noted above.

MedWatch 3500A Reporting Guidelines
In addition to completing appropriate patient demographic and suspect medication information, the report should include the following information within the Event Description (section 5) of the MedWatch 3500A form:

- Protocol description (and number, if assigned)
- Description of event, severity, treatment, and outcome if known
- Supportive laboratory results and diagnostics
- Investigator’s assessment of the relationship of the adverse event to each investigational product and suspect medication

Follow-up Information
Additional information may be added to a previously submitted report by any of the following methods:

- Adding to the original MedWatch 3500A report and submitting it as follow-up
- Adding supplemental summary information and submitting it as follow-up with the original MedWatch 3500A form
- Summarizing new information and faxing it with a cover letter including patient identifiers (i.e. D.O.B. initial, patient number), protocol description and number, if assigned, brief adverse event description, and notation that additional or follow-up information is being submitted (The patient identifiers are important so that the new information is added to the correct initial report)

Occasionally Genentech may contact the reporter for additional information, clarification, or current status of the patient for whom and adverse event was reported. For questions regarding SAE reporting, you may contact the Genentech Drug Safety representative noted above or the MSL assigned to the study. Relevant follow-up information should be submitted to Genentech Drug Safety as soon as it becomes available and/or upon request.
MedWatch 3500A (Mandatory Reporting) form is available at
http://www.fda.gov/medwatch/getforms.html

Study Drug Relationship:
The investigator will determine which events are associated with the use of study drug. For reporting purposes, an AE should be regarded as possibly related to the use of pertuzumab if the investigator believes:

- There is a clinically plausible time sequence between onset of the AE and pertuzumab administration; and/or
- There is a biologically plausible mechanism for pertuzumab to cause or contribute to the AE; And
- The AE cannot be attributed solely to concurrent/underlying illness.

A serious treatment emergent adverse event (STEAE) is any sign, symptom or medical condition that emerges during pertuzumab treatment or during a post-treatment follow-up period that (1) was not present at the start of pertuzumab treatment and it is not a chronic condition that is part of the patient’s medical history, OR (2) was present at the start of pertuzumab treatment or as part of the patient’s medical history but worsened in severity

and/or frequency during therapy, AND that meets any of the following regulatory serious criteria:

- Results in death
- Is life-threatening
- Requires or prolongs inpatient hospitalization
- Is disabling
- Is a congenital anomaly/birth defect
- Is medically significant or requires medical or surgical intervention to prevent one of the outcomes listed above.

Assessing Causality:

All AEs and SAEs whether volunteered by the subject, discovered by study personnel during questioning, or detected through physical examination, laboratory test, or other means will be reported appropriately. Each reported AE or SAE will be described by its duration (i.e., start and end dates), regulatory seriousness criteria if applicable, suspected relationship to the { Pertuzumab, Trastuzumab} (see following guidance), and actions taken.

The event should be assessed to decide whether there is a reasonable possibility that pertuzumab caused or contributed to an adverse event. The following general guidance may be used.

<u>Yes:</u> if the temporal relationship of the clinical event to pertuzumab administration makes a causal relationship possible, and other drugs, therapeutic interventions or underlying conditions do not provide a sufficient explanation for the observed event.

<u>No:</u> if the temporal relationship of the clinical event to pertuzumab administration makes a causal relationship unlikely, or other drugs, therapeutic interventions or underlying conditions provide a sufficient explanation for the observed event.

Expected adverse events are those adverse events that are listed or characterized in the Package Insert or current Investigator Brochure.
Unexpected adverse events are those not listed in the Package Insert (P.I.) or current Investigator Brochure (I.B.) or not identified. This includes adverse events for which the specificity or severity is not consistent with the description in the P.I. or I.B. For example, under this definition, hepatic necrosis would be unexpected if the P.I. or I.B. only referred to elevated hepatic enzymes or hepatitis.

Procedures for Eliciting, Recording, and Reporting Adverse Events
Eliciting Adverse Events
A consistent methodology for eliciting AEs at all subject evaluation time points should be adopted. Examples of non-directive questions include:

- “How have you felt since your last clinical visit?”
- “Have you had any new or changed health problems since you were last here?”

Specific Instructions for Recording Adverse Events

Investigators should use correct medical terminology/concepts when reporting AEs or SAEs. Avoid colloquialisms and abbreviations.

a. Diagnosis vs. Signs and Symptoms

If known at the time of reporting, a diagnosis should be reported rather than individual signs and symptoms (e.g., record only liver failure or hepatitis rather than jaundice, asterixis, and elevated transaminases). However, if a constellation of signs and/or symptoms cannot be medically characterized as a single diagnosis or syndrome at the time of reporting, it is ok to report the information that is currently available. If a diagnosis is subsequently established, it should be reported as follow-up information.

b. Deaths

All deaths that occur during the protocol-specified AE reporting period (see Section 5.1.2), regardless of attribution, will be reported to the appropriate parties. When recording a death, the event or condition that caused or contributed to the fatal outcome should be reported as the single medical concept. If the cause of death is unknown and cannot be ascertained at the time of reporting, report “Unexplained Death”.

c. Preexisting Medical Conditions

A preexisting medical condition is one that is present at the start of the study. Such conditions should be reported as medical and surgical history. A preexisting medical condition should be re-assessed throughout the trial and reported as an AE or SAE only if the frequency, severity, or character of the condition worsens during the study. When reporting such events, it is important to convey the concept that the preexisting condition has changed by including applicable descriptors (e.g., “more frequent headaches”).

d. Hospitalizations for Medical or Surgical Procedures

Any AE that results in hospitalization or prolonged hospitalization should be documented and reported as an SAE. If a subject is hospitalized to undergo a medical or surgical procedure as a result of an AE, the event responsible for the procedure, not the procedure itself, should be reported as the SAE. For example, if a subject is hospitalized to undergo coronary bypass surgery, record the heart condition that necessitated the bypass as the SAE.

Hospitalizations for the following reasons do not require reporting:

- Hospitalization or prolonged hospitalization for diagnostic or elective surgical procedures for preexisting conditions
- Hospitalization or prolonged hospitalization required to allow efficacy measurement for the study or
- Hospitalization or prolonged hospitalization for scheduled therapy of the target disease of the study.

e. Pregnancy

If a female subject becomes pregnant while receiving investigational therapy or within 90 days after the last dose of study drug, a report should be completed and expeditiously submitted to the Genentech, Inc. Follow-up to obtain the outcome of the pregnancy should also occur.

Abortion, whether accidental, therapeutic, or spontaneous, should always be classified as serious, and expeditiously reported as an SAE. Similarly, any congenital anomaly/birth defect in a child born to a female subject exposed to the {study drug} should be reported as an SAE.

f. Post-Study Adverse Events

The investigator should expeditiously report any SAE occurring after a subject has completed or discontinued study participation if attributed to prior {study drug} exposure. If the investigator should become aware of the development of cancer or a congenital anomaly in a subsequently conceived offspring of a female subject who participated in the study, this should be reported as an SAE.

g. Reconciliation

The Sponsor agrees to conduct reconciliation for the product. Genentech and the Sponsor will agree to the reconciliation periodicity and format, but agree at minimum to exchange monthly line listings of cases received by the other party. If discrepancies are identified, the Sponsor and Genentech will cooperate in resolving the discrepancies. The responsible individuals for each party shall handle the matter on a case-by-case basis until satisfactory resolution.

h. AEs of Special Interest (AESIs)

AEs of Special Interest are defined as a potential safety problem, identified as a result of safety monitoring of the Product

The [Pertuzumab] **Events of Special Interest are:**

- Cardiac event of special interest
- LV ejection fraction decrease
- LV dysfunction
- Heart Failure
- LVEF decrease

The [Trastuzumab] **Events of Special Interest are:**

- Cardiac Events

For questions related to safety reporting, contact:
Roche/Genentech Drug Safety
Pertuzumab Safety Monitor
Tele: 1-888-835-2555
Fax: (650) 225-4682 or or (650) 225-5288

Study Close-Out

Any literature articles that are a result of the study should be sent to Genentech. Copies of such

reports should be mailed to the assigned Clinical Operations contact for the study:

18.0 INFORMED CONSENT PROCEDURES

Before protocol-specified procedures are carried out, consenting professionals will explain full details of the protocol and study procedures as well as the risks involved to participants prior to their inclusion in the study. Participants will also be informed that they are free to withdraw from the study at any time. All participants must sign an IRB/PB-approved consent form indicating their consent to participate. This consent form meets the requirements of the Code of Federal Regulations and the Institutional Review Board/Privacy Board of this Center. The consent form will include the following:

1. The nature and objectives, potential risks and benefits of the intended study.
2. The length of study and the likely follow-up required.
3. Alternatives to the proposed study. (This will include available standard and investigational therapies. In addition, patients will be offered an option of supportive care for therapeutic studies.)
4. The name of the investigator(s) responsible for the protocol.
5. The right of the participant to accept or refuse study interventions/interactions and to withdraw from participation at any time.

Before any protocol-specific procedures can be carried out, the consenting professional will fully explain the aspects of patient privacy concerning research specific information. In addition to signing the IRB Informed Consent, all patients must agree to the Research Authorization component of the informed consent form.

Each participant and consenting professional will sign the consent form. The participant must receive a copy of the signed informed consent form.

19.0 REFERENCES

1. Siegel R, Naishadham D, Jemal A: Cancer statistics, 2012. CA Cancer J Clin 62:10-29, 2012
2. Slamon DJ, Clark GM, Wong SG, et al: Human breast cancer: correlation of relapse and survival with amplification of the HER-2/neu oncogene. Science 235:177-82, 1987
3. Robidoux A, Tang G, Rastogi P, et al: Evaluation of lapatinib as a component of neoadjuvant therapy for HER2+ operable breast cancer: NSABP protocol B-41. ASCO Annual Meeting 2012
4. Sinicrope FA, Foster NR, Yoon HH, et al: Association of obesity with DNA mismatch repair status and clinical outcome in patients with stage II or III colon carcinoma participating in NCCTG and NSABP adjuvant chemotherapy trials. J Clin Oncol 30:406-12, 2012
5. Cooley S, Burns LJ, Repka T, et al: Natural killer cell cytotoxicity of breast cancer targets is enhanced by two distinct mechanisms of antibody-dependent cellular cytotoxicity against LFA-3 and HER2/neu. Exp Hematol 27:1533-41, 1999

6. Izumi Y, Xu L, di Tomaso E, et al: Tumour biology: herceptin acts as an anti-angiogenic cocktail. Nature 416:279-80, 2002

7. Slamon DJ, Leyland-Jones B, Shak S, et al: Use of chemotherapy plus a monoclonal antibody against HER2 for metastatic breast cancer that overexpresses HER2. N Engl J Med 344:783-92, 2001

8. Romond EH, Perez EA, Bryant J, et al: Trastuzumab plus adjuvant chemotherapy for operable HER2-positive breast cancer. N Engl J Med 353:1673-84, 2005

9. Piccart-Gebhart MJ, Procter M, Leyland-Jones B, et al: Trastuzumab after adjuvant chemotherapy in HER2-positive breast cancer. N Engl J Med 353:1659-72, 2005

10. Marty M, Cognetti F, Maraninchi D, et al: Randomized phase II trial of the efficacy and safety of trastuzumab combined with docetaxel in patients with human epidermal growth factor receptor 2-positive metastatic breast cancer administered as first-line treatment: the M77001 study group. J Clin Oncol 23:4265-74, 2005

11. Pegram MD, Lopez A, Konecny G, et al: Trastuzumab and chemotherapeutics: drug interactions and synergies. Semin Oncol 27:21-5; discussion 92-100, 2000

12. Baselga J, Tripathy D, Mendelsohn J, et al: Phase II study of weekly intravenous recombinant humanized anti-p185HER2 monoclonal antibody in patients with HER2/neu-overexpressing metastatic breast cancer. J Clin Oncol 14:737-44, 1996

13. Cobleigh MA, Vogel CL, Tripathy D, et al: Multinational study of the efficacy and safety of humanized anti-HER2 monoclonal antibody in women who have HER2-overexpressing metastatic breast cancer that has progressed after chemotherapy for metastatic disease. J Clin Oncol 17:2639-48, 1999

14. Vogel CL, Cobleigh MA, Tripathy D, et al: Efficacy and safety of trastuzumab as a single agent in first-line treatment of HER2-overexpressing metastatic breast cancer. J Clin Oncol 20:719-26, 2002

15. Burstein HJ, Harris LN, Marcom PK, et al: Trastuzumab and vinorelbine as first-line therapy for HER2-overexpressing metastatic breast cancer: multicenter phase II trial with clinical outcomes, analysis of serum tumor markers as predictive factors, and cardiac surveillance algorithm. J Clin Oncol 21:2889-95, 2003

16. Jahanzeb M, Mortimer JE, Yunus F, et al: Phase II trial of weekly vinorelbine and trastuzumab as first-line therapy in patients with HER2(+) metastatic breast cancer. Oncologist 7:410-7, 2002

17. Yamamoto D, Iwase S, Kitamura K, et al: A phase II study of trastuzumab and capecitabine for patients with HER2-overexpressing metastatic breast cancer: Japan Breast Cancer Research Network (JBCRN) 00 Trial. Cancer Chemother Pharmacol 61:509-14, 2008

18. Seidman AD, Berry D, Cirrincione C, et al: Randomized phase III trial of weekly compared with every-3-weeks paclitaxel for metastatic breast cancer, with trastuzumab for all HER-2 overexpressors and random assignment to trastuzumab or not in HER-2 nonoverexpressors: final results of Cancer and Leukemia Group B protocol 9840. J Clin Oncol 26:1642-9, 2008

19. Seidman AD, Fornier MN, Esteva FJ, et al: Weekly trastuzumab and paclitaxel therapy for metastatic breast cancer with analysis of efficacy by HER2 immunophenotype and gene amplification. J Clin Oncol 19:2587-95, 2001

20. Perez EA, Vogel CL, Irwin DH, et al: Multicenter phase II trial of weekly paclitaxel in women with metastatic breast cancer. J Clin Oncol 19:4216-23, 2001

21. Tedesco KL, Thor AD, Johnson DH, et al: Docetaxel combined with trastuzumab is an active regimen in HER-2 3+ overexpressing and fluorescent in situ hybridization-positive metastatic breast cancer: a multi-institutional phase II trial. J Clin Oncol 22:1071-7, 2004

22. Burstein HJ, Kuter I, Campos SM, et al: Clinical activity of trastuzumab and vinorelbine

in women with HER2-overexpressing metastatic breast cancer. J Clin Oncol 19:2722-30, 2001

23. O'Shaughnessy JA, Vukelja S, Marsland T, et al: Phase II study of trastuzumab plus gemcitabine in chemotherapy-pretreated patients with metastatic breast cancer. Clin Breast Cancer 5:142-7, 2004

24. Schaller G, Fuchs I, Gonsch T, et al: Phase II study of capecitabine plus trastuzumab in human epidermal growth factor receptor 2 overexpressing metastatic breast cancer pretreated with anthracyclines or taxanes. J Clin Oncol 25:3246-50, 2007

25. Franklin MC, Carey KD, Vajdos FF, et al: Insights into ErbB signaling from the structure of the ErbB2-pertuzumab complex. Cancer Cell 5:317-28, 2004

26. Nahta R, Hung MC, Esteva FJ: The HER-2-targeting antibodies trastuzumab and pertuzumab synergistically inhibit the survival of breast cancer cells. Cancer Res 64:2343-6, 2004

27. Junttila TT, Akita RW, Parsons K, et al: Ligand-independent HER2/HER3/PI3K complex is disrupted by trastuzumab and is effectively inhibited by the PI3K inhibitor GDC-0941. Cancer Cell 15:429-40, 2009

28. Scheuer W, Friess T, Burtscher H, et al: Strongly enhanced antitumor activity of trastuzumab and pertuzumab combination treatment on HER2-positive human xenograft tumor models. Cancer Res 69:9330-6, 2009

29. Agus DB, Gordon MS, Taylor C, et al: Phase I clinical study of pertuzumab, a novel HER dimerization inhibitor, in patients with advanced cancer. J Clin Oncol 23:2534-43, 2005

30. Portera CC, Walshe JM, Rosing DR, et al: Cardiac toxicity and efficacy of trastuzumab combined with pertuzumab in patients with [corrected] human epidermal growth factor receptor 2-positive metastatic breast cancer. Clin Cancer Res 14:2710-6, 2008

31. Baselga J, Gelmon KA, Verma S, et al: Phase II trial of pertuzumab and trastuzumab in patients with human epidermal growth factor receptor 2-positive metastatic breast cancer that progressed during prior trastuzumab therapy. J Clin Oncol 28:1138-44, 2010

32. Cortes J, Fumoleau P, Bianchi GV, et al: Pertuzumab monotherapy after trastuzumab-based treatment and subsequent reintroduction of trastuzumab: activity and tolerability in patients with advanced human epidermal growth factor receptor 2-positive breast cancer. J Clin Oncol 30:1594-600, 2012

33. Baselga J, Cortes J, Kim SB, et al: Pertuzumab plus trastuzumab plus docetaxel for metastatic breast cancer. N Engl J Med 366:109-19, 2012

34. Swain SM, Tang G, Geyer CE, et al: NSABP B-38: Definitive analysis of a randomized adjuvant trial comparing dose-dense (DD) AC→paclitaxel (P) plus gemcitabine (G) with DD AC→P and with docetaxel, doxorubicin, and cyclophosphamide (TAC) in women with operable, node-positive breast cancer. J Clin Oncol 30 (suppl;abstr LBA1000), 2012

35. Datko FM, D'Andrea G, Dickler MN, et al: Phase II study of pertuzumab, trastuzumab, and weekly paclitaxel in patients with HER2-overexpressing metastatic breast cancer (MBC). J Clin Oncol 30 suppl 27, 2012

36. Costelloe CM, Chuang HH, Madewell JE, et al: Cancer response criteria and bone metastases: RECIST 1.1, MDA and PERSIST. J OF Cancer 1: 80-92, 2010

37. Wahl RL, Jacene H, Kasamon Y, et al: From RECIST to PERSIST: Evolving considerations for PET response criteria in solid tumors. JNM 50: 122S-150S, 2009

38. Juweid ME, Cheson BD: Positron-Emission tomography and assessment of cancer therapy. NEJM 354:496-506, 2006

39. Wahl RL, Zasadny K, Helvie M, et al: Metabolic monitoring of breast cancer chemohormonotherapy using positron emission tomography: initial evaluation. J Clin Onco 11: 2101-2111, 1993

40. Schelling M, Avril N, Nahring J, et al: Positron emission tomography using (18F)-fluorodeoxy-d-glucose for monitoring primary chemotherapy in breast cancer. J Clin Oncol 18: 1689-1695,2000

41. Kolesnikov-Gauthier H, Vanlemmens L, Baranzelli M, et al: Predictive value of neoadjuvant chemotherapy failure in breast cancer using FDG-PET after the first course. Breast Cancer Res Treat 131: 517-525, 2012

42 Burris HA, 3rd, Rugo HS, Vukelja SJ, et al: Phase II study of the antibody drug conjugate trastuzumab-DM1 for the treatment of human epidermal growth factor receptor 2 (HER2)-positive breast cancer after prior HER2-directed therapy. J Clin Oncol 29:398-405, 2011

43. Krop IE, LoRusso P, Miller KD, et al: A phase II study of trastuzumab emtansine in patients with human epidermal growth factor receptor 2-positive metastatic breast cancer who were previously treated with trastuzumab, lapatinib, an anthracycline, a taxane, and capecitabine. J Clin Oncol 30:3234-41, 2012

44. Hurvitz SA, Dirix L, Kocsis J, et al: Phase II Randomized Study of Trastuzumab Emtansine Versus Trastuzumab Plus Docetaxel in Patients With Human Epidermal Growth Factor Receptor 2-Positive Metastatic Breast Cancer. J Clin Oncol 31:1157-63, 2013

45. Verma S, Miles D, Gianni L, et al: Trastuzumab emtansine for HER2-positive advanced breast cancer. N Engl J Med 367:1783-91, 2012

46.Wildiers H, Kim SB, Gonzalez-Martin A, et al. T-DM1 for HER2-Positive MBC: Primary Results From TH3RESA, a Phase 3 Study of T-DM1 vs Treatment of Physician's Choice. European Society of Mecical Oncolgy 2013 (LBA # 15).

47. Blackwell KL, Burstein HJ, Storniolo AM, et al. Randomized study of Lapatinib alone or in combination with trastuzumab in women with ErbB2-positive, trastuzumab-refractory metastatic breast cancer. J Clin Oncol. 2010;28(7):1124-1130.

48. Clinical Trials.gov. http://clinicaltrials.gov/show/NCT01120184. March 6, 2013.

49. Seidman A, Hudis C, Pierri MK, et al: Cardiac dysfunction in the trastuzumab clinical trials experience. J Clin Oncol 20:1215-21, 2002

50. Perez EA, Rodeheffer R: Clinical cardiac tolerability of trastuzumab. J Clin Oncol 22:322-9, 2004

51. Lenihan D, Suter T, Brammer M et al. Pooled analysis of cardiac safety in patients with cancer treated with pertuzumab. Ann Oncol 23(3):791-800, 2012.

52. Ellis PA, Barrios CH, Eiermann W, Toi M, Im YH, Conte PF, Martin M, Pienkowski T, Pivot XB, Burris HA, Strasak A, Patre M, Perez EA: Phase III, randomized study of trastuzumab emtansine (T-DM1) ± pertuzumab (P) vs trastuzumab + taxane (HT) for first-line treatment of HER2-positive MBC: Primary results from the MARIANNE study. ASCO 2015. Abstract 507.

20.0 APPENDICES

Appendix A ECOG Performance Status Scale (attached separately)

Appendix B Diarrhea and Rash Management (attached separately)

Appendix C Hartford Alliance Protocol Addendum (attached separately)
