# Supplementary material for: Efficacy and Safety of Gemcitabine With Trastuzumab and Pertuzumab After Prior Pertuzumab-Based Therapy Among Patients With Human Epidermal Growth Factor Receptor 2–Positive Metastatic Breast Cancer: A Phase 2 Clinical Trial
Source: JAMA Netw Open. 2019 Nov 27;2(11):e1916211. doi: 10.1001/jamanetworkopen.2019.16211 (PMC6902832; doi:10.1001/jamanetworkopen.2019.16211)
Supplement: Supplement 2. — eFigure. Study Flow Diagram [file jamanetwopen-2-e1916211-s002.pdf]

Supplementary Online Content

Iyengar NM, Smyth LM, Lake D, et al. Efficacy and safety of gemcitabine with trastuzumab and pertuzumab after prior pertuzumab-based therapy among patients with human epidermal growth factor receptor 2–positive metastatic breast cancer: a phase 2 clinical trial. *JAMA Netw Open*. 2019;2(11):e1916211. doi:10.1001/jamanetworkopen.2019.16211

eFigure. Study Flow Diagram

This supplementary material has been provided by the authors to give readers additional information about their work.

© 2019 Iyengar NM et al. *JAMA Network Open.*

eFigure. Study Flow Diagram

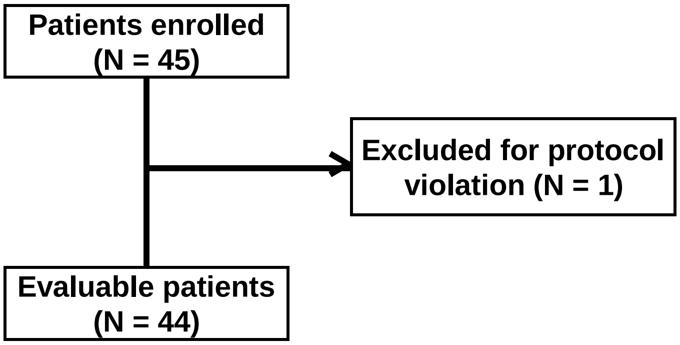

© 2019 Iyengar NM et al. *JAMA Network Open.*
